# Supplementary material for: A new Middle Jurassic diplodocoid suggests an earlier dispersal and diversification of sauropod dinosaurs
Source: Nat Commun. 2018 Jul 24;9:2700. doi: 10.1038/s41467-018-05128-1 (PMC6057878; doi:10.1038/s41467-018-05128-1)
Supplement: Supplementary file 1 — Supplementary Information [file 41467_2018_5128_MOESM1_ESM.pdf]

Supplementary Information for

A new Middle Jurassic diplodocoid suggests an earlier dispersal and diversification of sauropod dinosaurs

Xing Xu[1*‡], Paul Upchurch[2‡], Philip D. Mannion[3‡], Paul M. Barrett[4], Omar R. Regalado-Fernandez[2], Jinyou Mo[5], Jinfu Ma[6], Hongan Liu[7]

[1]Key Laboratory of Evolutionary Systematics of Vertebrates, Institute of Vertebrate Paleontology & Paleoanthropology, Chinese Academy of Sciences, Beijing 100044, People's Republic of China.

[2]Department of Earth Sciences, University College London, Gower Street, London, WC1E 6BT, United Kingdom.

[3]Department of Earth Science and Engineering, Imperial College London, South Kensington Campus, London, SW7 2AZ, United Kingdom.

[4]Department of Earth Sciences, Natural History Museum, Cromwell Road, London, SW7 5BD, United Kingdom.

[5]Natural History Museum of Guangxi, Nanning, 530012, Guangxi, People's Republic of China.

[6]Lingwu National Geopark Administration, Lingwu, 750400, Ningxia, People's Republic of China.

[7]Lingwu Historic Relic Administration, Lingwu, 750400, Ningxia, People's Republic of China.

*Correspondence and requests for materials should be addressed to X.X. (email: xu.xing@ivpp.ac.cn).

‡Equally contributing authors.

This PDF file includes:

Supplementary Notes

Supplementary Note 1: Abbreviations

1.1. Institutions

1.2. Morphology

1.3. Other

Supplementary Note 2: Age of the Yanan Formation

Supplementary Note 3: Additional morphological description

Supplementary Note 4: Phylogenetic analyses

4.1. *Lingwulong* specimen-level OTUs

4.2. Dataset choice and modifications

4.3. Phylogenetic assumptions

4.4. Phylogenetic results (main dataset)

4.5. Phylogenetic results (subsidiary dataset)

Supplementary Note 5: The status of *Lingwulong* as the earliest known, and first confirmed Asian, diplodocoid

5.1. Putative early diplodocoids

5.2. Putative early neosauropods

5.3. Putative East Asian diplodocoids

Supplementary Note 6: Biogeographic analyses

6.1. Taxon ages

6.2. Time-calibrated phylogeny

6.3. Taxon geographic ranges

6.4. Dispersal multiplier matrices

6.5. Analyses, results, and interpretations

6.6. Additional analysis and results

Supplementary Note 7: Chinese sauropod biogeography, diversity, and sampling

Supplementary Figures 1 to 15

Supplementary References

Supplementary Notes

Supplementary Note 1: **Abbreviations**

1.1. Institutions

CM, Carnegie Museum of Natural History, Pittsburgh, USA; FHPR, Utah Field House of Natural History State Park Museum, Vernal, USA; IGPAS, Institute of Paleobiology, Georgian Academy of Sciences, Republic of Georgia; IVPP, Institute of Vertebrate Paleontology and Paleoanthropology, Beijing, People's Republic of China; LM, Lingwu Museum, Lingwu, Ningxia Hui Autonomous Region, People's Republic of China; LGP, Lingwu Geopark, Lingwu, Ningxia Hui Autonomous Region, People's Republic of China; MACN, Museo Argentino de Ciencias Naturales 'B. Rivadavia', Buenos Aires, Argentina; MUCPv, Museo de Geología y Paleontología de la Universidad Nacional del Comahue, Neuquén, Argentina; NHMUK, Natural History Museum, London, UK; OUMNH, Oxford University Museum of Natural History, Oxford, UK; PMU, Palaeontological Museum, University of Uppsala, Sweden.

1.2. Morphology

We use the terminology for vertebral laminae and fossae proposed by Wilson [1] and Wilson et al. [2], respectively. The abbreviations associated with these terms, used in this study, are: CPOF, centropostzygapophyseal fossa; CPRF, centroprezygapophyseal fossa; CPRL, centroprezygapophyseal lamina; EPRL, epipophyseal-prezygapophyseal lamina; PODL, postzygodiapophyseal lamina; SDF, spinodiapophyseal fossa; SPDL, spinodiapophyseal lamina; SPOL, spinopostzygapophyseal lamina; SPRL, spinoprezygapophyseal lamina; TPRL, interprezygapophyseal lamina.

1.3. Other

EAIH, East Asian Isolation Hypothesis; FAD, First Appearance Datum; LAD, Last Appearance Datum; MRCA, most recent common ancestor; MPT, most parsimonious tree; OTU, operational taxonomic unit.

Supplementary Note 2: **Age of the Yanan Formation**

The *Lingwulong* specimens were found in the Yanan Formation, Ningxia Hui Autonomous Region, northwest China (Supplementary Fig. 1). This formation outcrops in the Chinese provinces of Ningxia, Shanxi, Gansu, and Inner Mongolia, within the large sedimentary Ordos Basin [3, 4, 5, 6, 7, 8, 9, 10, 11]. The Yanan Formation represents a series of swampy and fluviolacustrine facies that varies in thickness from ~120–450 m, and is composed of greyish-white sandstones, grey-black and greyish-brown silt- and mudstones, and

intercalated coal beds, with conglomerates in the basal part [4, 5, 6, 10, 12]. The formation has been divided into four [10, 13] or five members [4]. The member yielding the *Lingwulong* specimens is currently uncertain. The sediments at the excavation site comprise a ~20m thickness of lacustrine mudstones overlying multiple coal beds: *Lingwulong* specimens occur in yellowish-grey and greyish-brown massive mudstones, with occasional cross-bedding structures. Given that the only member described as having mudstones overlying coal beds is member 4 [4], we provisionally suggest that *Lingwulong* comes from the upper part of the Yanan Formation.

To date, no radiometric constraints have been obtained for the Yanan Formation itself (but see below), and its age has been estimated on the basis of biostratigraphy and its relationships to under- and overlying units. The Yanan Formation has yielded plant macrofossils, pollen, spores, bivalves, gastropods, ostracods, conchostracans, and rare dinosaur body fossils and tracks, a biota that is consistent with either a late Early Jurassic or early Middle Jurassic age [3, 5, 6, 9, 11, 14]. For example, conchostracans in the Yanan Formation include *Palaeoleptoestheria, Triglypta,* and *Euestheria* [6], an assemblage that is indicative of a Middle Jurassic age (potentially pre-Callovian in the case of the latter genus [14, 15]).

According to Wang et al. [8], the Yaopo Formation in western Beijing and the Biennia Formation in western Liaoning, can be correlated with the Yanan Formation of the Ordos Basin. This study notes that the Yaopo and Beipiao formations overlie volcanic sequences in western Liaoning that have been dated at 191–180 Ma [16, 17, 18]. This is consistent with the late Toarcian age for the Yanan Formation proposed by Wang et al. [8], and certainly suggests that it is no older than this. However, this constraint does not rule out an Aalenian or younger age.

The Yanan Formation overlies the Fuxian Formation and underlies the Zhiluo Formation, with these relationships apparently retained across a wide geographic area in northwest China [4, 8, 10, 19]. There is disagreement concerning the conformability/disconformability of these contacts. In particular, the Fuxian-Yanan contact is shown as conformable in table 1 of Wang et al. [8], but listed as disconformable by Johnson et al. [4], Chen [5], Chen and Yang [19], and Tanner et al. [10]. Similarly, the Yanan-Zhiluo contact is regarded as conformable by Chen [5], but disconformable by Johnson et al. [4], Wang et al. [8], and Tanner et al. [10]. It is difficult to determine to what extent this uncertainty reflects differences in interpretation, versus genuine variation in the nature of the contacts depending on which part of this geographically extensive sedimentary sequence is examined.

There is consensus that the underlying Fuxian Formation is Early Jurassic in age [4, 5, 8, 10, 15, 19]. This unit could be older than the Pliensbachian [5, 19], though more recent studies and some biostratigraphic data suggest a Pliensbachian–early Toarcian age [8, 15].

Biostratigraphic studies have universally concluded that the overlying Zhiluo Formation is Middle (often late Middle) Jurassic in age (e.g., [7, 20]), and attempts have been made to narrow its dating to Stage level. Such estimates include: Aalenian (table 1 in ref. [8]), Aalenian–early Bajocian [5], Aalenian–Bajocian [9],

Bathonian [21], and Bathonian–Callovian [10]. Furthermore, the Zhiluo Formation is itself overlain by the Anding Formation in the Ordos Basin, with the latter unit being estimated to be latest Middle Jurassic (typically Callovian) by most studies [5, 8, 12, 19, 21], though Tanner et al. [10] regarded it as early Late Jurassic in age. If these age estimates are approximately correct, then it would be unlikely for the Yanan Formation to be much older than late Toarcian given the radiometric constraints and the age of the Fuxian Formation, and equally improbable for it to be younger than Bajocian given the requirement to fit the overlying Zhiluo, and potentially also Anding, formations into the late Middle Jurassic.

Recent work on the tectonic history of the Ordos Basin also supports an Aalenian–Bajocian age for the Yanan Formation [22, 23]. These studies indicate that the Ordos Basin passed through a tectonically 'quiet' extensional phase during the Early and early Middle Jurassic, a time when most of the coal-bearing deposits were laid down. A key transition occurred approximately 168 Ma [23] as a result of a shift towards a compressional phase that produced uplift, regression of lakes, and erosion (as marked by the conglomeratic deposits of the Zhiluo Formation). Thus, the Yanan Formation is interpreted as being deposited during the earlier extensional phase and so predates the shift from extension to compression at ~168 Ma.

In summary, the foregoing studies have concluded that the Yanan Formation is either late Early Jurassic [8, 9, 19] or early Middle Jurassic [3, 6, 10, 11, 12] in age. In terms of specific stage estimates, Chen [5] suggested that the Yanan Formation was Toarcian–Aalenian in age. This was narrowed to late Toarcian by Wang et al.[8]. However, Li et al. (table 7 in ref. [14]) proposed that the Yanan Formation spans the Aalenian–Bajocian based on conchostracan biostratigraphy, and this is consistent with the views of Tanner et al. [10]. Clearly, considerable additional work is required to produce agreement and provide narrower constraints on the age of the Yanan Formation, and indeed many other geological units in the Jurassic of northwest China. One problem with dating these units is the lack of intercalated marine sequences [7, 10]. Moreover, the Ordos Basin generally lacks volcanic units that can be used to constrain the ages of Jurassic sedimentary series. Here, we conservatively and provisionally regard the age of *Lingwulong* as somewhere within the late Toarcian–Bajocian: this gives a midpoint age of ~174.15 Ma based on the International Commission on Stratigraphy Chronostratigraphic Chart 2016 [24].

Supplementary Note 3: **Additional morphological description**

A full monographic description of *Lingwulong* is planned as the next step in this project, but here we provide an augmented description, with additional figures that focus on key synapomorphies and autapomorphies.

Cranial material includes the skull roof, occiput, and braincase. As in dicraeosaurids [25, 26], the frontals are co-ossified along the midline (Fig. 1, Supplementary Fig. 2). The dorsal orbital margins are thickened dorsoventrally and marked by an irregular series of protrusions and pits

(an autapomorphy). Prefrontals project anterolaterally and curve strongly laterally towards their free anterior tips, another autapomorphy of *Lingwulong* (Fig. 1, Supplementary Fig. 2). Supratemporal fenestrae are large and open dorsolaterally. The frontoparietal suture is located midway between the anterior and posterior margins of the supratemporal fenestrae, as in advanced dicraeosaurids and some diplodocids [27]. There is a hook-like, posteroventrally-directed process on the main body of the squamosal, as is also seen in a mild form in the diplodocid *Kaatedocus* and prominently in dicraeosaurids [25, 26, 27]. In lateral view, the squamosal ventral process projects anteroventrally, implying the presence of an anteroventrally oriented quadrate and a long slit-like lower temporal opening, as is typical for diplodocoids [25, 28]. The postorbital ventral process has a subtriangular transverse cross-section. This process is not strongly compressed anteroposteriorly, which is highly unusual for a eusauropod [29], though it also occurs in the rebbachisaurid *Limaysaurus* (MUCPv-205). As in dicraeosaurids [25], the sagittal crest on the supraoccipital is prominent and plate-like in *Lingwulong* (Fig. 1, Supplementary Fig. 2). *Suuwassea* [30], *Amargasaurus* (MACN N-15), and *Lingwulong* (but not *Dicraeosaurus* or other sauropods) possess a deep slot-like fossa on the occiput, lateral to the proatlantal facets. The convex occipital condyle is unusually wide relative to its dorsoventral height (width:height ratio = 1.54), although this might have been exaggerated by crushing. The paroccipital processes are directed laterally in dorsal view. In occipital view, these processes extend horizontally in their medial halves, but more distally they curve strongly ventrolaterally (Fig. 1, Supplementary Fig. 2).

Basal tubera are prominent, unlike the derived state in rebbachisaurids such as *Limaysaurus* and *Nigersaurus*, where they are very reduced [25]. In ventral view, the long-axes of the free distal tips of the basal tubera are directed anteromedially (Fig. 1, Supplementary Fig. 2), rather than transversely or posteromedially as in other sauropods [27]. Above the basipterygoid process, the left otosphenoidal ridge bears the broken base of a dorsoventrally flattened ‘leaf’-like process, a derived state in dicraeosaurids [26, 28]. In lateral view, with the skull roof horizontal, the broken bases of the basipterygoid processes project anteroventrally at approximately 45º, as in flagellicaudatans and some titanosaurs [25, 28, 31]. Unlike most sauropods, but similar to dicraeosaurids [26, 28], there is a deep slot-like channel on the ventral midline of the basisphenoid of *Lingwulong*, extending between the bases of the basipterygoid processes (Fig. 1, Supplementary Fig. 2).

A ridge extends dorsally from the broken base of the parasphenoid rostrum to the ventral margin of the large opening for cranial nerve I (Olfactory). The openings for cranial nerve II (Optic) are separated from each other on the anterior midline by the abutting anterior margins of the orbitosphenoids. The crista antotica extends dorsally in front of the large opening for cranial nerve V, and dorsally expands laterally as the capitate process that separates the posterodorsal part of the orbital cavity from the supratemporal opening. This process is mediolaterally long

relative to its dorsoventral height (length:height ratio *c*. 5.0) in *Lingwulong*, compared to a ratio of 2.0–3.0 in other sauropods, such as *Dicraeosaurus* and *Giraffatitan* [32].

IVPP V23704 preserves 29 teeth in a ‘U’-shaped arc (Fig. 1, Supplementary Fig. 3), suggesting that the whole set either became detached from the jaw as a unit, or that the jaw elements decayed, leaving the more resistant teeth in situ. This is probably a reliable indication that the jaw margin was not square, unlike those of most other diplodocoids [25, 28, 33]. We tentatively identify these as dentary teeth, based on distolabially facing wear facets: this would mean that there were ~15 dentary teeth in life. Wear facets resemble those of diplodocoids, being typically set at ≥45º to the apicobasal axis [25]. Teeth are mildly prognathous, increase slightly in size towards the jaw symphysis, and lack serrations. As in other diplodocoids, tooth crowns do not overlap each other in an imbricate arrangement [28, 29]. Unlike typical sauropod spatulate crowns, those of *Lingwulong* are nearly symmetrical in labial view and do not curve lingually towards their apices (Supplementary Fig. 3). There are very faint apicobasally-directed grooves on the labial surfaces close to the distal margins. As in other diplodocoids and advanced titanosauriforms [28, 29, 31], the lingual surface of the crown is convex mesiodistally, creating an elliptical horizontal cross-section (Supplementary Fig. 3).

The true number of cervical, dorsal, and caudal vertebrae in *Lingwulong* is unknown, although we estimate a count of 11–12 dorsals. Cervical centra are opisthocoelous and short anteroposteriorly compared to their height. As in dicraeosaurids [25], the ventral surfaces of the cervical centra are deeply excavated anteriorly to produce a pair of pneumatic fossae separated by a prominent midline keel. A deep lateral pneumatic opening is present on anterior cervical centra, but is shallow or absent in more posterior cervical and dorsal vertebrae. Although the absence of deep lateral pneumatic openings in presacral centra is plesiomorphic for sauropods [28, 29], this also occurs as a derived reversal in dicraeosaurids [25]. In *Lingwulong* cervical centra, there is a small accessory fossa located just posteroventral to the main lateral pneumatic fossa. This feature has previously only been reported in diplodocids [25, 26, 27], but it is also variably present in the dicraeosaurid *Amargasaurus* (MACN N-15). Cervical pre- and postzygapophyses have flat articular surfaces and do not project significantly beyond the anterior and posterior margins of the centrum. There is an unusual ridge-like projection on the lateral surface of each prezygapophyseal process, near its base (Fig. 2, Supplementary Fig. 4). There is no epipophyseal-prezygapophyseal lamina (EPRL) within the spinodiapophyseal fossa (SDF). Neural spines are bifurcated from the middle cervicals posteriorly to approximately dorsal 5. Cervical neural spines lack the extreme elongation seen in derived dicraeosaurids [25, 34, 35]. In lateral view, there is a deep ‘U’-shaped notch between the prezygapophyses and anterior spine margin, and the angle between the PODL and SPOL is ~90º (Fig. 2, Supplementary Fig. 4); both features are characteristic of dicraeosaurids [35, 36]. The metapophyses of *Lingwulong* are directed dorsally to create a deep and transversely narrow ‘V’-shaped notch, an intermediate condition between the broadly open ‘V’- or ‘U’-shaped notches in most sauropods with bifid presacrals, and the extremely tall and narrow notches (in which the metapophyses often curve medially

towards their summits) that occur in advanced dicraeosaurids (e.g., *Amargasaurus* and *Dicraeosaurus*) [35]. The metapophyses of *Lingwulong* are unusual in bearing smooth dorsolaterally facing subcircular 'facets' at their summits (Fig. 2, Supplementary Fig. 4). *Lingwulong* possesses the derived shortened cervical ribs seen in other diplodocoids [25, 26, 27].

Anterior dorsal centra are strongly opisthocoelous, but from approximately dorsal 4 onwards they become amphicoelous, as in other diplodocoids [26] (Fig. 2, Supplementary Fig. 5). Dorsal centra are subcircular in transverse cross-section throughout their length, and are generally taller and wider than they are long anteroposteriorly. A deep CPRF is present between the CRPLs, below the TPRL and above the neural canal. The transverse wall of bone between this CPRF and the CPOF is thin anteroposteriorly, as also seen in *Dicraeosaurus* [34] and *Haplocanthosaurus* (CM 572). The transverse processes of the anterior and middle dorsals are directed upwards at ~30º, but become horizontal in posterior dorsals. Unlike other taxa, *Lingwulong* possesses a small anterodorsally-directed process on the anterior margins of the anterior dorsal diapophyses near their distal ends. SPRLs are well-developed in anterior dorsals, but from dorsal 6 onwards these are 'captured' by the diapophyses so that they form anterior SPDLs. Middle dorsals possess a posterior SPDL, but this is lost in the most posterior ones. There is a well-developed hyposphene-hypantrum system on middle and posterior dorsals (Supplementary Fig. 5). Posterior dorsal neural spines are tall (spine:centrum height ~2.0), with spine height increasing towards the sacrum. Metapophyses in anterior dorsals are unusual in being twisted along their length, such that the SPOLs project posteriorly at their base and laterally towards the summit (Supplementary Fig. 5). In middle dorsals, the spines have a 'paddle'-shaped morphology, in which their lateral margins gradually flare outwards as they approach the transversely rounded summit (Fig. 2, Supplementary Fig. 5), a derived condition seen in the middle and posterior dorsals of rebbachisaurids and dicraeosaurids [25, 26, 34]. In *Lingwulong*, the posterior-most dorsal spines more closely resemble those of non-diplodocoids (e.g., turiasaurs and non-titanosaurian macronarians) in possessing well-developed subtriangular aliform processes projecting laterally from the summit. None of the anterior dorsal ribs display the apomorphic 'plank-like' morphology of advanced macronarians [29, 31]. Pneumatic fossae (near the capitulum) occur in some dorsal ribs in *Lingwulong.*

The sacrum consists of five fused vertebrae. Sacral centra lack lateral pneumatic fossae and are mildly amphicoelous. Sacral centra 3 and 4 are the most constricted transversely. Neural spines 2–4 are coalesced (Supplementary Fig. 6), as in most flagellicaudatans [28]. Each spine terminates in a rugose summit, with aliform processes supported by a stout spinodiapophyseal lamina (Supplementary Fig. 6). Sacral ribs 2–5 fuse distally to form a 'sacricostal yoke' [29], but only sacral ribs 2–4 actually contribute to the dorsal margin of the acetabulum.

Caudal centra are shallowly amphicoelous and subcircular in transverse cross-section (Fig. 2, Supplementary Fig. 7). They lack lateral pneumatic fossae below the base of the rib, unlike some rebbachisaurids, diplodocines, and titanosauriforms [25, 26, 27, 36]. The ventral surfaces of the caudal centra are transversely rounded in *Lingwulong*, rather than being excavated between ventrolateral ridges as occurs in some diplodocines and titanosaurs [28]. In anterior caudals, the low rounded SPRLs extend onto the lateral surface of each spine (Supplementary Fig. 7), a derived state observed in flagellicaudatans [29]. Unlike other flagellicaudatans, however, SPRLs do not contact SPOLs. Anterior caudal neural spines are tall (spine:centrum height ~2.0), curving slightly posteriorly towards their summits. These spines are subtriangular in horizontal cross-section, with an acute anterior prespinal rugosity and wider posterior margin formed by the SPOLs. The first 11 caudal neural spines are unusual in having subtriangular facet-like areas on their lateral surfaces that extend from the summit to approximately spine mid-height. The ventral tips of these facets are expanded laterally to form small processes, resembling the triangular projections seen in some rebbachisaurids [26]. The anterior-most caudal ribs of *Lingwulong* have the wing-like structure present in most diplodocoids [28]: the anterior surface is deeply excavated and the dorsolateral corner forms a distinct projection. In most diplodocoids, however, the latter projection is a low 'shoulder'-like region, whereas in *Lingwulong* it is a prominent dorsally-directed prong. This 'prong' is not regarded as an autapomorphy of *Lingwulong* because it potentially occurs in a few other putative diplodocoids (e.g., *Haplocanthosaurus* FHPR 1106). The neural arches of middle caudal vertebrae are located centrally, rather than being anteriorly shifted as in titanosauriforms [28, 31]. Anterior chevrons are bridged proximally (Supplementary Fig. 8), as in most non-eusauropod sauropods and flagellicaudatans [37]. The haemal canal occupies approximately 25% of total chevron length in anterior elements, the plesiomorphic condition [31, 37]. Mid-tail chevrons are 'forked' (Supplementary Fig. 8), as also occurs in non-neosauropod eusauropods and flagellicaudatans [29, 37].

The scapula has a strongly expanded proximal plate and a long blade with a widened distal end (although it is not racket-shaped as occurs in rebbachisaurids [26]; Fig. 2). With the scapulocoracoid long-axis extending horizontally, the dorsal margin of the proximal scapular expansion lies at a higher level than that of the coracoid (Supplementary Fig. 9). The prominent acromial (deltoid) ridge is at ~90º to the long-axis of the blade. The glenoid is not deflected medially as in *Apatosaurus* and somphospondylans [29]. In cross-section, the blade is 'D'-shaped, as is typical for all eusauropods, apart from early forms (such as *Shunosaurus*) and some advanced titanosaurs [29]. In the coracoid (Fig. 2), the notch anterior to the glenoid is weakly developed and the glenoid region does not expand markedly laterally, unlike those of some *Camarasaurus*-like macronarians [38]. The anterodorsal margin of the coracoid is rounded rather than square, the latter being a derived state that occurs in some diplodocoids and titanosaurs [28].

The proximal end of the humerus is strongly convex transversely (Fig. 2, Supplementary Fig. 8). The deltopectoral crest extends to mid-length and does not expand medially across the anterior surface of the humerus, unlike the derived state seen in many titanosaurs [29, 31]. As in diplodocids [27], the humerus is twisted so that, with the long-axis through the proximal end extending transversely, the long-axis through the distal end is directed posterolaterally. The lateral margin of the humerus, in anterior view, is moderately concave proximodistally. Ulnae possess a triradiate proximal end with a deep radial fossa, as also observed in other sauropods (Supplementary Fig. 8) [28, 29] . The proximal anteromedial process of the ulna lacks the concave profile present in several titanosauriforms [31]. The radius has a 'D'-shaped proximal end with a flattened rugose articular surface. Unlike several titanosauriforms [31], there is no prominent ridge extending proximodistally along the posterior shaft. The distal end of the radius is convex and, in anterior view (Fig. 2, Supplementary Fig. 8), moderately beveled so that it faces laterodistally.

The ilium (Fig. 2) has a reduced ischial articulation, rounded dorsal profile, and lacks a brevis fossa, as is typical of sauropods [28]. The preacetabular process is subtriangular in outline, the plesiomorphic condition that contrasts with the rounded profile in titanosauriforms [28]. Unfortunately, this preacetabular process has been pushed inwards by post-mortem crushing in the one clearly exposed specimen, so it is not possible to determine whether it was directed anteriorly (as in non-neosauropod eusauropods) or anterolaterally (as in neosauropods) [28, 29]. As in several other flagellicaudatans (e.g., *Dicraeosaurus*, *Diplodocus* [26]), the pubis bears a prominent ambiens process immediately anterior to the iliac articulation (Fig. 2): however, this process tapers in dorsoventral width towards its tip in *Lingwulong*, rather than being 'hooked'. The ischial articulation is ~33% of pubis length. A large elliptical obturator foramen pierces the pubis near its proximoposterior corner. In lateral view, the distal end of the pubis is moderately expanded anteroposteriorly relative to the rest of the shaft. The ischium is relatively slender. In lateral view (Fig. 2), the iliac peduncle of the ischium lacks the derived constriction ('neck') seen in most rebbachisaurids [25, 26]. There is a long, low, rounded ridge extending along the lateral surface of the proximal shaft, bounding the ventral margin of a moderately deep groove. This muscle insertion has this morphology in most sauropods, but is a sharp ridge in rebbachisaurids and a prominent bulge (lacking a groove) in titanosauriforms [26, 31]. The distal end surface of the ischium retains the plesiomorphic subtriangular profile seen in most non-macronarian sauropods [28], yet the conjoined distal ends are co-planar (an apomorphy observed in macronarians and rebbachisaurids [28, 29] that has not previously been seen in a flagellicaudatan).

The femur (Fig. 2, Supplementary Fig. 9) resembles those of other sauropods, although the proximal head is directed medially, rather than dorsomedially [28]. Its fourth trochanter is a low rounded ridge on the posteromedial margin of the femoral shaft, at approximately mid-length. The tibia has a transversely widened proximal end, a derived state seen in neosauropods [29], with the cnemial crest directed laterally (Supplementary Fig. 9). Its distal end has a reduced medial

malleolus, exposing the astragalus posteriorly, as in most sauropods [29]. The astragalus has a transversely and anteroposteriorly convex ventral surface and tapered medial projection. Its ascending process extends to the posterior margin of the astragalus, a derived state characteristic of neosauropods [29]. The fibular facet faces laterally, rather than posterolaterally as occurs in many diplodocoids [25]. The ventral margin of this facet also projects laterally, so that it would have underlain the distal end of the fibula when articulated. As in other sauropods, there is a foramen on the posterior part of the medial surface of the ascending process.

Supplementary Note 4: **Phylogenetic analyses**

4.1. *Lingwulong* specimen-level OTUs

In order to test the hypothesis that the specimens at the Lingwu Geopark site represent a monospecific assemblage, we have scored six of the more informative sets of specimens separately and incorporated them as OTUs in our main data matrix (see below). These OTUs are as follows:

'Lingwulongskull' – This includes character scores for the holotypic braincase found in quarry II (LDM V001a) and the two sets of teeth. The better-preserved set (IVPP V23704), probably from the dentaries, was found in quarry III. A less well-preserved set of teeth was found in close association with the braincase in Quarry II. The current OTU therefore combines braincase and dental character scores because of the specimen association and because the two sets of teeth are essentially identical in morphology.

'Lingwulongcervicals' – This is based on three articulated middle or middle-posterior cervical vertebrae (field nos. I 106–108), a single middle cervical (field no. III 131), and two other similar cervical vertebrae (unnumbered, in IVPP). These three sets of cervical vertebrae are combined into a single OTU because they are very similar in overall morphology, and share at least one autapomorphy (the roughened ridge-like structure on the dorsolateral surface of the base of the prezygapophyseal process).

'Lingwulongantdorsal' – This is based on a series of six articulated anterior-middle dorsal vertebrae (II 148–153).

'Lingwulongpartialskela (LGP V003)' – This is a partial skeleton comprising the four posterior-most dorsal vertebrae (II 026–029), the sacrum (II 018–022), left and right ilia (II 025 [left] and 017 [right]), and the first two caudal vertebrae (II 023 and 024), all in articulation. In addition, three articulated middle dorsal vertebrae (II 031–033) and six thoracic ribs (II 001, 003, 004, 006, 011 and 016) lie close to the articulated portion and their positions suggest that they probably belong to the same individual.

'Lingwulongpartialskelb' – This is a partial skeleton comprising the four posterior-most dorsal vertebrae (II 037–041), the sacrum (II 042–046), and the first caudal vertebra (II 047) in articulation. There is also an articulated partial right hind limb preserving the femur, tibia, and astragalus (II 084–086), with the proximal

end of the femur close to the sacrum. We consider this hind limb to be part of the same individual as the dorsal-sacrum-caudal series represented by II 037–047. This postcranial skeleton is the paratype (LGP V001b) and is potentially the same individual as the holotypic partial skull which was found close to the dorsal vertebrae.

'Lingwulongpartialskelc' – This is a sacrum and pelvis (I 111–113, 073) and associated and partially articulated series of 25 anterior and middle caudal vertebrae (I 072 [first caudal vertebra] to I 044 [last preserved middle caudal vertebra]). The sacral and pelvic elements are largely obscured by matrix. Caudal vertebrae 1–14 are articulated, as are the remaining 11 middle caudal vertebrae: although slightly separated, the series of middle caudal vertebrae extends along approximately the same line as the more anterior elements. There are no caudal vertebrae labelled I 058–054, indicating a potential break in the series, and there might also have been another break between caudal I 053 and 052.

'Lingwulongtotal' – As well as the above six specimen-level OTUs, we have scored a composite OTU based on all of the specimens available in the three quarries.

4.2. Dataset choice and modifications

We have scored the six specimen-level OTUs, and 'Lingwulongtotal', for the data matrix of Rauhut et al. [39]. The original version of this matrix comprises 370 characters scored for 72 sauropodomorph taxa. We have chosen to use this matrix because it is the most up-to-date version of the series of datasets produced by Carballido and colleagues [40, 41, 42, 43, 44], and includes scores for an unnamed Late Jurassic diplodocid from Argentina. This dataset is suitable for our study because it samples a phylogenetically and spatiotemporally wide array of sauropodomorph taxa, and thus gives our new taxon the freedom to cluster anywhere within known sauropod diversity. It therefore provides a suitable test of our hypothesis that *Lingwulong* represents a dicraeosaurid diplodocoid. The ref. [39] dataset is also one of the largest available for sauropods (N.B., the dataset of ref. [27] is larger, but this is because it is a specimen-level analysis focusing on diplodocids, and thus is not suitable for assessing the relationships of *Lingwulong* more broadly within Sauropoda). Here, we term our version of the Rauhut et al. matrix our 'main dataset'. We have also scored 'Lingwulongtotal' for the data matrix of ref. [45], which we term our 'subsidiary dataset'. The latter is a diplodocoid-focused species-level dataset derived from the specimen-level analysis of ref. [27]. As such, ref. [45] does not provide a fair test of the wider relationships of *Lingwulong*, but it could potentially yield insights into the placement of this taxon within Diplodocoidea.

The Rauhut et al. [39] dataset has been modified by the addition of five characters pertaining to the skull and cervical and anterior dorsal vertebrae that we believe are relevant to diplodocoid relationships. These additional characters have been placed at the end of the character list as numbers 371–375, as follows:

371. Exoccipital, dorsolateral margin in posterior view – spur of bone curves dorsolaterally and then ventrolaterally to form the dorsomedial margin of the posttemporal fenestra: absent (0); present (1) (new

character; see ref. [27]: fig. 4). This character is related to another character (C41 in ref. [39]) that scores for the presence/absence of a contribution of the parietal to the margin of the posttemporal fenestra (see also ref. [29]). Many diplodocids have the parietal excluded from this fenestra by an exoccipital-squamosal contact. This is a derived state that is linked to the presence of the curving process from the exoccipital. However, taxa such as *Amargasaurus* and *Lingwulong* demonstrate that it is possible to have this process while simultaneously possessing a parietal contribution to the margin of the posttemporal fenestra. Essentially, it seems that this process from the exoccipital developed in flagellicaudatans, but only contacts the squamosal in a subset of these taxa (i.e., mainly diplodocids). In order to capture all of the character states present, it is therefore necessary to introduce the additional character employed here.

372. Exoccipital – small, deep, horizontally oriented groove immediately lateral to each of the proatlantal facets: absent (0); present (1) (new character).

373. Postaxial cervical centra – small fossa on posteroventral corner of lateral surface: absent (0); shallow, anteroposteriorly elongate fossa present, posteroventral to main lateral pneumatic opening (1) [26, 46].

374. Middle cervical neural spines – angle between PODL and SPOL in lateral view: acute less than 85º (usually close to 45º) (0); 85º or more (usually 90º) (1) [35].

375. Posterior cervical and anterior dorsal bifid neural spines – morphology of metapophyses in anterior view: widely diverging (0) narrow, parallel to converging (1) [35]. *Lingwulong*, which could be regarded as possessing a state that is intermediate between 0 and 1, is provisionally scored with state 1 here because its condition appears to be closer to the derived state.

We also corrected two scoring errors in ref. [39] dataset, pertaining to *Amygdalodon*. This taxon was scored with state 1 for character numbers 67 (absence/presence of large lateral projection on otosphenoidal ridge [=crista prootica]) and 71 (basal tubera breadth:occipital condyle breadth) by ref. [39]. However, the only cranial material known for *Amygdalodon* is teeth [47]. In our version of the data matrix, therefore, these characters were corrected to '?' for *Amygdalodon*.

The main data matrix, in TNT format, is given in Supplementary Data 1.

4.3. Phylogenetic assumptions

In all analyses of the main data set, the following characters were treated as ordered (i.e., 'additive' in TNT): 12, 58, 95, 96, 102, 106, 108, 115, 116, 119, 120; 145, 152, 163, 213, 216, 232–235, 252, 256, 298, 299, and 301 (N.B., this list is incorrect in Rauhut et al. [39] – the correct list is provided in Carballido and Sander [40].

4.4. Phylogenetic results (main dataset)

Analysis with the six *Lingwulong* specimen-level OTUs. Note that in these analyses the composite OTU 'Lingwulongtotal' was excluded a priori. Analysis in TNT yielded 168 MPTs of length 1106 steps. The strict

consensus (Supplementary Fig. 10) shows that all six *Lingwulong* specimens form a polytomy with the other four dicraeosaurid genera (*Amargasaurus*, *Brachytrachelopan*, *Dicraeosaurus*, *Suuwassea*). While this does not, by itself, demonstrate that all the putative *Lingwulong* specimens belong to the same species, it is certainly consistent with such a proposal. Dicraeosaurids have not been reported from East Asia previously, so the presence of six dicraeosaurid specimens at the same locality is suggestive. Moreover, our phylogenetic dataset does not include any of the putative autapomorphies of *Lingwulong*: addition of these characters is likely to cause at least some of the specimen-level OTUs to cluster together. Thus, the array of probable associations, the repeated presence of autapomorphies in specimens sharing overlapping elements, and the lack of evidence for more than one sauropod taxon at the locality, all combine to support the view that the Lingwu Geopark has yielded a monospecific assemblage.

Analysis with the composite *Lingwulong* OTU ('Lingwulongtotal'). Note that in these analyses the six specimen-level OTUs for *Lingwulong* were excluded a priori. This analysis produced 12 MPTs of length 1107 steps. The strict consensus (Supplementary Fig. 11) shows a fully resolved Dicraeosauridae with *Lingwulong* located as the sister-taxon to *Amargasaurus*+(*Dicraeosaurus*+*Brachytrachelopan*). The agreement subtree (Supplementary Fig. 12) was generated in TNT via a posteriori deletion of the following OTUs: *Barapasaurus*, *Haplocanthosaurus priscus*, Calcareo diplodocid, *Histriasaurus*, *Rebbachisaurus*, *Bellusaurus*, *Chubutisaurus,* and *Argentinosaurus*.

GC values (Supplementary Fig. 11) indicate that this relationship is relatively strongly supported, having the sixth highest value (i.e., 46) out of 70 nodes (i.e., the 72 nodes in a fully resolved 73-taxon tree, excluding the two most basal nodes which are constrained by the designation of *Plateosaurus* as the outgroup). Indeed, the placement of *Lingwulong* within Dicraeosauridae is better supported than several other widely accepted relationships within Sauropoda, such as the monophyly of Diplodocoidea, Diplodocidae, Rebbachisauridae, and Macronaria, and is stronger than any of the nodes within the latter clade. Supplementary Data 15 summarizes the synapomorphies supporting the placement of *Lingwulong* within Diplodocoidea, Flagellicaudata, Dicraeosauridae, and 'higher dicraeosaurids' (i.e., *Lingwulong* plus the *Amargasaurus*+*Dicraeosaurus* clade, to the exclusion of *Suuwassea*). Based on the main dataset, there are 22 synapomorphies supporting the placement of *Lingwulong* within Diplodocoidea or a less inclusive clade: 11 of these have individual consistency indices of 1.0 (i.e., they display no homoplasy), and a further six only occur convergently in a small number of neosauropods. We conclude, therefore, that there is strong support for the position of *Lingwulong* within Dicraeosauridae.

Analysis with *Lingwulong* constrained to lie outside Neosauropoda. This analysis produced three MPTs of length 1117 steps (i.e., 10 steps longer than the unconstrained MPTs). A Templeton's test shows that these constrained MPTs are not a statistically worse explanation of the available data ($p = 0.114–0.140$), indicating that current data could support a non-neosauropod placement of *Lingwulong*. However, our modified Rauhut et al. [39] dataset does not contain all of the character data relevant to the position of *Lingwulong* within Diplodocoidea or a less inclusive clade. For example, the derived strongly dorsally deflected cervical

prezygapophyses seen in *Lingwulong, Dicraeosaurus, Amargasaurus,* etc. have not yet been incorporated as a character in our dataset. Similarly, further study of the field associations of *Lingwulong* specimens should yield additional information on limb proportions that are relevant to diplodocoid affinities (e.g., forelimb:hind limb ratio). Thus, we anticipate that the evidence for the placement of *Lingwulong* as a dicraeosaurid will strengthen in the future as a result of further work.

4.5. Phylogenetic results (subsidiary dataset)

'Lingwulongtotal' was scored for the diplodocoid-focused dataset of Tschopp and Mateus [45]. This dataset originally comprised 489 characters for 35 taxa. We added two additional characters, numbers 490 and 491, which are identical to numbers 371 and 372 in our main dataset (see above: N.B., the other three characters added to the Rauhut et al. matrix [i.e., numbers 373–375] were not added to this subsidiary dataset because they were already present). This produced a dataset comprising 36 OTUs and 491 characters (this is available in TNT format in Supplementary Data 2).

This subsidiary dataset was analysed in TNT using the protocol outlined above. The following characters were ordered, as in Tschopp and Mateus (2017)[45]: 5, 58, 63, 86, 88, 95, 114, 116, 117, 130, 131, 144, 157, 158, 170, 172, 198, 230, 280, 292, 295, 305, 310, 314, 346, 357, 359, 370, 372, 391, 392, 399, 407, 414, 426, 439, 442, and 478. Under equal weights parsimony, this yielded 12 MPTs of length 1587 steps. The strict consensus of these 12 MPTs is shown in Supplementary Fig. 14. This includes a non-traditional result in which apatosaurines form the sister-taxon to other flagellicaudatans. This is not the result of adding *Lingwulong* to this dataset: such a topology also occurs when the original Tschopp and Mateus (2017)[45] dataset is analyzed using equal weights. Consequently, these authors preferred to use implied weighting that, as occurs here, recovers a more traditional topology with Apatosaurinae and Diplodocinae as sister-taxa, to the exclusion of Dicraeosauridae (Tschopp and Mateus: fig. 78)[45]. Application of implied weighting to our subsidiary dataset recovered a single MPT of length 128.31735 steps (Supplementary Fig. 15) (N.B., application of the theoretically superior Extended Implied Weighting approach [52] produced an identical single MPT of length 120.07339). In the equally weighted MPTs (Supplementary Fig. 14), *Lingwulong* is placed in a trichotomy with *Amargasaurus* and *Brachytrachelopan*, with *Dicraeosaurus* and *Suuwassea* as successively more distant outgroups within Dicraeosauridae. When Implied Weighting and Extended Implied Weighting are applied, *Lingwulong* is placed as the sister-taxon of a clade containing *Dicraeosaurus, Amargasaurus,* and *Brachytrachelopan* (Supplementary Fig. 15), as was found by the analyses of our main dataset.

A full comparison of our results with those of Tschopp and Mateus [45], and investigation of the root causes of the topological differences in terms of character state distributions, lies beyond the scope of the current study. Nevertheless, it is worth noting that the addition of *Lingwulong* has a significant impact on the topology of the Tschopp and Mateus tree when either type of Implied Weighting is applied. For example, the two *Galeamopus* species no longer form a monophyletic group; *Amphicoelias* no longer clusters within *Brontosaurus,* but is instead the sister-taxon to other apatosaurines; and *Dicraeosaurus*, rather than

Brachytrachelopan, is the sister-taxon to *Amargasaurus*. It is likely that the addition of *Lingwulong* has affected some character polarities and homoplasy scores, which in turn have changed the support for the relationships among other clades such as Diplodocidae. This issue deserves further detailed analysis.

Many of the character states that support the placement of *Lingwulong* within Dicraeosauridae, Flagellicaudata, and Diplodocoidea are essentially the same as those identified by the analysis of our main dataset (see Supplementary Data 15). However, character mapping based on the Implied Weights tree (Supplementary Fig. 15) and the subsidiary dataset reveals some additional support. There are 23 synapomorphies that support the placement of *Lingwulong* within Diplodocoidea or a less inclusive clade, with 10 of these being unequivocal. Of the remaining 13 synapomorphies, 10 show only small amounts of homoplasy, with convergence normally involving other neosauropods.

Supplementary Note 5: **The status of *Lingwulong* as the earliest known, and first confirmed Asian, diplodocoid**

In this study, we propose that *Lingwulong* is the earliest diplodocoid, the earliest neosauropod, and the first confirmed diplodocoid from Asia. However, several previous works have identified other specimens as the earliest members of these clades or as Asian diplodocoids. Many of these specimens are fragmentary, a problem that has contributed to the uncertainties surrounding their relationships. Even if such identifications are correct, their status as the earliest member of a given sauropod clade is overturned by the probable late Toarcian–Bajocian age estimated for *Lingwulong*. Thus, in order to set the discovery of *Lingwulong* in the wider context of sauropod evolution, we briefly review other Middle and early Late Jurassic specimens that are potentially early diplodocoids or neosauropods, as well as the few specimens identified previously as Asian diplodocoids.

5.1. Putative early diplodocoids

Three sauropod specimens, all from the United Kingdom, have previously been proposed as representing Middle Jurassic diplodocoids. These are: '*Cetiosaurus*' *glymptonensis* from the late Bathonian, composed of nine middle caudal centra (OUMNH J.13750–J.13758 [fig. 3 in ref. [53]]); NHMUK PV R1967, from the Callovian, consisting of a series of 10 distal caudal centra [54]; and the holotypic partial skeleton of *Cetiosauriscus stewarti* (NHMUK PV R3078), from the Callovian [28, 55, 56]. The diplodocoid affinities of these specimens were reviewed recently by Whitlock [25] and Mannion et al. [26], who concluded that there was little compelling evidence for these identifications. The arguments used by Whitlock and Mannion et al. to support this view will not be recapitulated here, except to note that '*Cetiosaurus*' *glymptonensis* and NHMUK PV R1967 are very incomplete and lack clear diplodocoid synapomorphies, and *Cetiosauriscus* has been placed outside of Neosauropoda by most phylogenetic analyses prior to 2012 (e.g., ref. [35]: though see below). Mannion et al. [26] therefore concluded that the earliest evidence for Diplodocinae (and so also

diplodocoids as a whole) comes from the middle Oxfordian of Abkhazia, western Republic of Georgia (IPGAS D-9 [57], fig. 2 in ref. [57]). Unfortunately, this Georgian material is also very incomplete (a single anterior caudal centrum): thus, even though subsequent studies have accepted its identification as the earliest diplodocine (e.g., ref. [39]), the specimen tells us little concerning the early phases of diplodocoid diversification and morphological evolution.

Since the reviews by Whitlock [25] and Mannion et al. [26], a larger and more up-to-date dataset compiled by Tschopp et al. [27] has resurrected the possibility that *Cetiosauriscus* is a diplodocoid. The equally weighted analyses of Tschopp et al. [27] (figs. 114, 115 in ref. [27]) placed *Cetiosauriscus stewarti* within Diplodocoidea, closer to Flagellicaudata than Rebbachisauridae. However, in this analysis, and that in which implied weighting was applied, a traditional Macronaria is paraphyletic with respect to Diplodocoidea (i.e., Titanosauriformes is more closely related to the latter clade than is *Camarasaurus*). Such a topology has not been supported by any previous phylogenetic analysis of Sauropoda (with the possible exception of Upchurch [58], in which titanosaurs and diplodocoids were sister-taxa to the exclusion of *Camarasaurus* and Brachiosauridae). The unusual topology found by Tschopp et al. [27] suggests that the emphasis on diplodocid taxa, and characters relevant to that clade, resulted in an under-sampling of the characters needed for accurate reconstruction of wider sauropod phylogeny. If correct, this suggests that the placement of *Cetiosauriscus* within Diplodocoidea should be treated with caution. Moreover, when implied weighting was used, *Cetiosauriscus* was placed outside of Neosauropoda by Tschopp et al. [27] (figs. 116, 117 in ref. [27]). Nevertheless, Tschopp et al. [27] did identify several character states that potentially support inclusion of *Cetiosauriscus* within Diplodocoidea. Some of these features (e.g., the relatively short forelimb) were cited by Berman and McIntosh [55] as diagnostic of 'Diplodocidae' (now equivalent to Diplodocoidea), and some are new character states such as a foramen on the dorsal surface of metatarsal I in *Cetiosauriscus* and several other diplodocoids. Thus, while we remain skeptical about the placement of *Cetiosauriscus stewarti* within Diplodocoidea, there is some evidence in support of this view. This English taxon is in need of thorough re-description and re-evaluation (a project being undertaken by PU and PDM). If *Cetiosauriscus stewarti* is a diplodocoid, its Callovian age and relative completeness would shed light on the initial phases of this clade's evolution, yet *Lingwulong* is stratigraphically earlier and much more completely known.

5.2. Putative early neosauropods

Various Middle Jurassic body fossils and several tracks have been identified as those of macronarians, and are potential candidates for the title of earliest known neosauropod. These taxa include *Abrosaurus* from China, *Atlasaurus* from Morocco, *Lapparentosaurus* from Madagascar, and the putative titanosaur tracks at Ardley in the United Kingdom [28, 40, 59, 60], but these identifications are far from universally accepted [31, 60, 61, 62, 63]. These proposals were reviewed recently by D'Emic [60] and Mannion et al. [31], who concluded that most of this Middle Jurassic material pertained to non-neosauropods, indeterminate sauropods, or non-sauropods. We therefore refer the reader to these two works, and focus instead on providing a brief update based on new information that has been presented since 2013.

Abrosaurus is known from the lower Shaximiao Formation (Bajocian) of China [64], and has been tentatively identified as a macronarian (e.g., ref. [28]). Given its early age and the fact that it includes a well-preserved skull, this taxon could provide important insights into the initial phases of macronarian diversification. However, *Abrosaurus* requires detailed re-evaluation before its true significance can be established. One problem is that *Abrosaurus* has been only briefly described, with little evaluation of detailed character data derived from recent phylogenetic analyses. This severely limits both the number and accuracy of character state scores that can be assessed for this taxon. Moreover, the phylogenetic significance of some of the 'macronarian' features of *Abrosaurus* can be challenged. For example, *Abrosaurus* appears to possess the derived enlarged external naris that was proposed as a macronarian synapomorphy by Wilson and Sereno [65], but it is conceivable that this feature evolved in non-neosauropod eusauropods and was lost in diplodocoids as a result of extreme narial retraction [61, 66].

Atlasaurus is based on an associated partial skull and postcranial skeleton from the Guettioua Formation (Bathonian) of Morocco, and was considered to be 'brachiosaurid-like' by Monbaron et al. [67]. Moreover, *Atlasaurus* was placed in a clade (with *Jobaria* and *Bellusaurus*) that formed the sister-taxon to other macronarians in the phylogenetic analysis of Upchurch et al. [28]. However, updated scores for *Euhelopus* resulted in this cluster of taxa being positioned outside of Neosauropoda [63]. Subsequent phylogenetic analyses of titanosauriform relationships by D'Emic [60] and Mannion et al. [31] confirm the placement of *Atlasaurus* as a non-neosauropod eusauropod rather than a macronarian or brachiosaurid (see also refs. [61, 68, 69]).

Lapparentosaurus is based on the remains of several disarticulated juvenile/subadult sauropods from the Isalo III Formation (late Bathonian) of Madagascar. Bonaparte [70] initially considered this taxon to be a relatively 'primitive' sauropod, based on the rudimentary development of its neural spine laminae. In contrast, McIntosh [71] and Upchurch [58, 72] suggested that *Lapparentosaurus* might represent an early brachiosaurid, based on features such as the strong ventral deflection of the distal ischial shaft, which is also seen in *Giraffatitan* [73]. However, increasing instability in the position of *Lapparentosaurus* meant that it was regarded as a titanosauriform of uncertain affinities by Upchurch et al. [28], and it had to be pruned a priori from the analyses of Wilson and Upchurch [63]. More recently, detailed phylogenetic analyses have placed *Lapparentosaurus* outside of Neosauropoda, and this does not seem to be attributable merely to the presence of plesiomorphic character states resulting from its early ontogenetic stage [31, 74, 75, 76]. For the present, therefore, *Lapparentosaurus* does not provide a convincing example of a Middle Jurassic macronarian.

Day et al. [59, 77] identified a series of trackways from the Middle Jurassic (Bathonian) White Limestone Formation of Ardley, Oxfordshire, United Kingdom, as belonging to a titanosaur. This was based on the wide-gauge nature of these tracks (e.g., ref. [78]) and the observation that the manus prints did not include an impression of a large pollex claw. The latter could be the result of poor preservation or the claw being held above the surface of the substrate, but the same trackway horizon also preserves narrow-gauge sauropod tracks with a pollex claw mark. This, combined with geologic data indicating that the tracks were

all generated during a short time window (i.e., between two tides), suggests that the putative wide-gauge track maker genuinely lacked a pollex claw. Loss of manual phalanges, including the pollex claw, is a derived state that occurs in advanced titanosaurs such *as Alamosaurus* and *Opisthocoelicaudia* [31, 59, 77, 79, 80, 81]. If advanced titanosaurs were present in the Bathonian, then this would imply that several other neosauropod lineages had diverged from each other during or before the early Middle Jurassic. However, D'Emic [60] questioned the identification of the Ardley tracks as titanosaurs, noting that wide-gauge tracks could have been made by non-titanosaurian titanosauriforms. He also pointed out that the wide-gauge Ardley tracks lacked the usual heteropody (i.e., size differential between smaller manus and larger pes prints), which potentially meant that the tracks were not preserved completely. Moreover, Henderson [82] has argued that the width of a sauropod trackway might be more influenced by the body mass of the track maker than by its phylogenetic affinities, with individuals heavier than 12 tonnes tending to make wider-gauge trackways because of biomechanical requirements (see also ref. [83]). Here, we maintain that the Ardley wide-gauge trackways are most plausibly interpreted as those of titanosaurs, or at least somphospondylans, given the known morphological modifications for a wider stance in these taxa [78]. Nevertheless, we acknowledge that the Ardley trackways do not provide decisive evidence that neosauropods were present in the Bathonian.

One of the best candidates for the earliest true macronarian is *Bellusaurus* from the Shishugou Formation (Callovian or early Oxfordian) of China [84, 85, 86, 87]. This genus is known from cranial and postcranial remains from several individuals and has been described in detail [87]: we might therefore expect that its phylogenetic position should be established on the basis of a larger number of scorable character states. Yet, despite its relative completeness, this taxon has proved to be controversial. *Bellusaurus* was placed in a clade with *Atlasaurus* and *Jobaria* as the sister-taxon to all other macronarians by Upchurch et al. [28], but character state revisions in Wilson and Upchurch [63] resulted in these three genera shifting to a position outside Neosauropoda. In contrast, *Bellusaurus* consistently clusters within basal Macronaria in analyses based on the data sets created by Carballido and colleagues (e.g., refs. [40, 44]), and a similar position was supported recently on the basis of new cranial material [88]. Other recent analyses, however, have continued to place *Bellusaurus* outside Neosauropoda (e.g., ref. [61]: see also ref. [62]). This disagreement can only be settled by further taxon and character sampling, and careful revision of character constructions and state scores. Pending this further work, we note here that, even if *Bellusaurus* is eventually confirmed as a macronarian, its Callovian/Oxfordian age makes it substantially younger than *Lingwulong*.

Lastly, the stratigraphically oldest sauropod for which macronarian affinities appear secure is a late Oxfordian specimen from France. *Vouivria* [89], previously known informally as the 'French *Bothriospondylus*' or 'Damparis sauropod', has been universally regarded as a brachiosaurid titanosauriform since its original description (e.g., refs. [28, 31, 58, 60]).

5.3. Putative East Asian diplodocoids

A small number of fragmentary specimens have been identified as East Asian diplodocoids. These were reviewed by Upchurch and Mannion [90] and all were shown to lack compelling supporting evidence.

Upchurch and Mannion [90], however, described an anterior caudal vertebra (PMU R263) from the late Early Cretaceous of Shandong, China, which they identified as a diplodocid based on the results of phylogenetic analysis. This specimen possesses several potential diplodocid synapomorphies, including a deep lateral pneumatic opening below the base of the caudal rib, a wing-like caudal rib, and complex lamination of the neural spine. However, Whitlock et al. [91] noted that several of these features are more widespread among neosauropods (especially certain somphospondylans), and pointed out that the specimen possesses the camellate tissue structure characteristic of the anterior caudal vertebrae of advanced titanosaurs. The affinities of PMU R263 have not been tested via a phylogenetic analysis that incorporates the revised character state scores identified by Whitlock et al. [91], although Mannion et al. [31] commented that some of these features support a placement among lithostrotian titanosaurs. Here, therefore, we provisionally accept the view of Whitlock et al. [91] that PMU R263 is not a diplodocoid, pending further analysis.

Recently, Shimizu et al. [92] reported a putative diplodocoid from the Berriasian–early Barremian Sao Khua Formation of Thailand. Given that the remains apparently include several parts of the axial and appendicular skeleton, this potentially represents strong evidence for the presence of diplodocoids in the Early Cretaceous of south-east Asia. However, it is currently impossible to evaluate this claim because the only information available is in a conference abstract [92]. All that can be said about this possible diplodocoid (at present) is that it has tall and bifid cervical neural spines, which is tantalizingly reminiscent of the condition seen in advanced dicraeosaurids.

In short, prior to the discovery of *Lingwulong*, there had been sporadic claims for the occurrence of diplodocoids in East Asia, all of which relate to specimens from the Early Cretaceous. With the possible exception of the Thai specimens reported by Shimizu et al. [92], all other such claims have been based on very incomplete specimens whose phylogenetic affinities are either disputed or shown to be non-diplodocoid. Thus, *Lingwulong* represents the first well-preserved confirmed diplodocoid from East Asia, and is certainly the first from the Jurassic of that region.

Supplementary Note 6: **Biogeographic analyses**

6.1. Taxon ages

The 65 taxa in the agreement subtree (and an additional four diplodocids, see below) have been dated using the Paleobiology Database (https://www.paleobiodb.org/) and Fossilworks Database (http://fossilworks.org/?a=home), as well as the primary literature. Some taxa, such as *Apatosaurus*, are known from multiple specimens at several stratigraphic horizons. Such taxa are given a stratigraphic range and so have different First Appearance Datum (FAD) and Last Appearance Datum (LAD) values. Other taxa are known from a single specimen, or multiple specimens from a single horizon, so that their ‘age range’ actually represents lack of temporal resolution rather than a true stratigraphic range. For example,

Dinheirosaurus, from the Late Jurassic of Portugal [31], is known from the Lourinha Formation (late Kimmeridgian–early Tithonian). Given that *Dinheirosaurus* is a singleton, its true stratigraphic age is a point lying somewhere within the listed range. Such taxa are given midpoint ages, as has been done in other recent macroevolutionary analyses (e.g., ref. [95]). Many taxon ages are listed in the literature in terms of Standard European Stages – these have been converted into absolute ages (in millions of years before present) using the International Commission on Stratigraphy Chronostratigraphic Chart 2016 (see above). The FADs, LADs, and midpoint ages for the 69 taxa used in the biogeographic analysis are given in Supplementary Data 3.

6.2. Time-calibrated phylogeny

The phylogenetic topology used in the biogeographic analyses (Supplementary Fig. 12) is based on the agreement subtree (see above): this is because phylogenetic biogeographic methods, such as BioGeoBEARS, require a fully resolved tree topology [94]. The Rauhut et al. [39] dataset only includes three diplodocid genera, all of which come from the Late Jurassic of North America. Because of our focus on the biogeography of diplodocoids, we have therefore augmented this topology by adding four diplodocid taxa based on the tree topology presented by Tschopp et al. [27]. The four additional taxa are *Tornieria*, *Dinheirosaurus*, and *Supersaurus* from the Late Jurassic of Africa, Europe, and North America, respectively, and *Leinkupal* from the Early Cretaceous of South America [31, 96, 97, 98]. (N.B., the relationships of these four additional taxa are also supported by Tschopp and Mateus [fig. 78 in ref. [45]], and also by both types of implied weighting analyses of the subsidiary dataset presented here [Supplementary Fig. 15]). The resulting 69-taxon tree was then calibrated against time using the taxon ages in Supplementary Data 3 (see section 6.1 above), using the R package strap [99, 100]. This was done via the Datephylo command, with a root length of 5 million years, and distributing adjacent zero-length branches using the 'equal' method (a modified version of the approach proposed by Brusatte et al. [101]). The resulting time-calibrated phylogeny is presented in newick format in Supplementary Data 4, and is shown in abbreviated form in Fig. 3 and in full in Supplementary Fig. 13. This time-calibrated topology is effectively a 'minimum age' tree: more sophisticated methods for dating phylogenies containing fossils have been proposed by Bapst [102], but are difficult to apply to datasets where most taxa are represented by point occurrences rather than genuine stratigraphic ranges (e.g., see the discussion in the supplementary information file for Poropat et al. [103]).

6.3. Taxon geographic ranges

BioGeoBEARS also requires a taxon geographic range file. Here, we have designated eight continental areas: A, Asia; E, Europe; F, Africa; I, India; M, Madagascar; N, North America; S, South America; and U, Australia. Each of the 69 taxa in the time-calibrated phylogeny has been scored for its presence/absence in each of these eight areas, generating a geographic range matrix that can be read by BioGeoBEARS (see Supplementary Data 5).

6.4. Dispersal multiplier matrices

A BioGeoBEARS analysis can be carried out solely using a time-calibrated tree and geographic range file; however, this would lack any information on the relative positions and connectedness of palaeogeographic areas. Adding such palaeogeographic data is likely to have a significant and beneficial effect on ancestral area estimation, especially if palaeogeography changes substantially during the evolution of the target taxa, and/or the taxa are large-bodied terrestrial forms that are unlikely to disperse across major marine barriers (e.g., ref. [103]). In BioGeoBEARS, the probability of dispersal between any given pair of areas can be controlled using one or more dispersal multiplier matrices [93]. Moreover, a series of dispersal multiplier matrices (each pertaining to a different time slice) can be used to represent how palaeogeographic areas fragmented and/or coalesced in response to plate tectonics, changes in sea level, etc.

In this analysis, we have employed the assumptions and analytical protocol presented in the supplementary information file for Poropat et al. [103]. Thus, for example, we assume that sauropods had a very low probability of dispersing directly across marine barriers, and that terrestrial dispersal involved crossing from one area to an immediately adjacent one. Below, we explain the small number of cases where our treatments of dispersal probabilities differ from those employed by Poropat et al. [103].

Temporal scope. Poropat et al. [103] analyzed data covering the Middle Jurassic to the end of the Cretaceous. Here, our oldest taxon is *Plateosaurus*, so we have extended the time range of our analyses to include the Norian to the Maastrichtian.

Geographic areas. Poropat et al. [103] scored taxa for seven continental-scale areas, with India and Madagascar combined into a single area. Here, we use eight areas, with India and Madagascar treated separately. This necessitates some adjustments to the dispersal multiplier matrices, especially to represent the separation of India from Madagascar during the Late Cretaceous. India and Madagascar were in contact throughout most of the Mesozoic until rifting between them commenced at approximately 88–87 Ma [104, 105]. From this time onwards, India moved rapidly away from Madagascar [105]. The exact point when India and Madagascar became fully separated by a marine barrier is not known, but the first oceanic crust between them is dated at around 84 Ma [106]. We have therefore modelled India and Madagascar as being in contact up to the mid-Coniacian (88.05 Ma), and fully separated during the Campanian and Maastrichtian (83.6–66 Ma). During the intervening break-up phase (i.e., late Coniacian–Santonian, 88.05–83.6 Ma), we have modelled these two areas as being in contact in our ‘relaxed’ dispersal multipliers and separated in our ‘harsh’ ones (see Poropat et al. [103] for further discussion of dealing with palaeogeographic uncertainties when creating dispersal multiplier matrices).

Europe-Asia dispersal probabilities during the Jurassic. A key issue in the debate over Jurassic East Asian isolation is the nature of putative marine barriers between Europe and Asia. During the Mesozoic, three main marine barriers potentially controlled Europe-Asia terrestrial dispersal: (1) the Mongol-Okhotsk Ocean between Siberia-Kazakhstan on the one hand and various East Asian continental blocks (e.g., Mongolia, Tarim, North and South China blocks, etc.) on the other; (2) the Uralian Sea (an epicontinental sea in the region of the Russian Platform, west of the Urals); and (3) the Turgai Sea (also an epicontinental

sea, but located further east than the Uralian Sea). There is now strong evidence that continental collision started to close the Mongol-Okhotsk Ocean during the Middle Jurassic (e.g., [104]), facilitating terrestrial dispersal between Central and East Asia from this time onwards (see review in Poropat et al. [103]). The Turgai Sea formed during the mid-Cretaceous (essentially from approximately the late Cenomanian onwards) and persisted into the Cenozoic [107]. However, there is evidence that fluctuating sea levels resulted in periodic connection and disconnection of European and Asian landmasses, across the Turgai Strait, during this period [107, 108]. The history of the Russian Platform/Uralian Sea marine barrier during the Cretaceous has also been discussed in detail by Baraboshkin et al. [107], and again it seems that this formed an ephemeral barrier to Europe/Asia terrestrial dispersal during the Early Cretaceous (see also the review in Poropat et al. [103]). The information summarized above was used by Poropat et al. [103] to develop detailed dispersal multiplier values for Europe-Asia during the Cretaceous. However, we have subsequently identified more precise data on the extent of the Russian Platform/Uralian Sea during the Middle and Late Jurassic that was not incorporated into the latter study. Below, therefore, we briefly outline this new information and describe how it affects the Jurassic dispersal multiplier matrices used here.

The Russian Sea, located in the region of Kostroma, Kursk, Moscow, Saratov, and Ulyanovsk, was connected to the peri-Tethyan Sea to the south, and northern Pechora Sea to the north, creating a north-south marine barrier between eastern Europe and Central Asia during the late Callovian–early Kimmeridgian [109, 110]. This epicontinental sea was bounded to the east by the Uralian uplands and northwest by Fenoscandia [111]. The seaway is believed to have been 100–200 m deep and to have extended between 40–70º N [111, 112]. These studies are consistent with the palaeocoastline reconstructions of Smith et al. [113], who depicted a marine barrier between Europe and Asia appearing for the first time in the Callovian. We therefore constrain Europe and Asia to be disconnected from each other during the late Callovian to the early Kimmeridgian in our dispersal multiplier matrices. Baraboshkin et al. [107] suggested that the Uralian Sea was breached by a Europe/Asia landbridge during the early Berriasian, and the Smith et al. [113] palaeocoastline maps show a gradual narrowing of the northernmost part of this sea during the Kimmeridgian and Tithonian, though it potentially remained intact as a north-south marine corridor. Given the current imprecision in the timing of the establishment of a Europe/Asia landbridge during the Kimmeridgian and Tithonian, we have treated this as 'uncertainty' in our dispersal multiplier matrices.

Poropat et al. [103] divided the Middle and Late Jurassic in to three time slices: (1) Bajocian–Oxfordian; (2) Kimmeridgian; and (3) Tithonian. The Europe/Asia dispersal multiplier values were set to 0.5 in the 'Starting' matrices for all three of these time slices, reflecting the perceived lack of information for the Middle and Late Jurassic. This meant that the Europe/Asia values were set to 1.0 in the relaxed, and 0.000001 in the harsh versions, respectively, of the dispersal multiplier matrices for these three time slices in Poropat et al. [103]. Here, we employ five dispersal multiplier matrices to cover the Late Triassic and Jurassic, as follows: (1) Norian–early Callovian (~227–164.8 Ma); (2) late Callovian–Oxfordian (164.8–157.3 Ma); (3) early Kimmeridgian (157.3–154.7 Ma); (4) late Kimmeridgian (154.7–152.1 Ma); and (5) Tithonian (152.1–145 Ma). In all versions of our dispersal multiplier matrices, the Europe/Asia values for the Norian–early

Callovian time slice are set to 1.0, whereas those for the late Callovian–Oxfordian and early Kimmeridgian time slices are set to 0.000001. The values for the late Kimmeridgian and Tithonian are set to 1 in the ‘relaxed’, and 0.000001 in the ‘harsh’, versions of our dispersal multiplier matrices (see below).

In this study, we use two different versions of our dispersal multiplier matrices – ‘relaxed’ and ‘harsh’. Essentially, palaeogeographic relationships that are strongly supported are identical in both sets of matrices, but those that are more uncertain in terms of the sequence and timing of events, are treated differently in the two types of matrix. Whenever such uncertainties occur, we make the assumption that the affected areas were in contact in our relaxed matrices and that they were not in contact in our harsh matrices (see ref. [103]). The relaxed and harsh dispersal multiplier matrices are presented in Supplementary Data 6 and 7, respectively. Supplementary Data 8 contains the ‘time periods file’ used to designate the starting ages (in millions of years before the LAD of the youngest taxon) of each of the 24 time slices.

6.5. Analyses, results, and interpretation

The results of the log likelihood ratio tests and AIC values are set out in Supplementary Data 16. The plots showing the ancestral area estimations for the best supported models (i.e., BAYAREALIKE+J for both the relaxed and harsh constraints) are presented in Supplementary Data 10 and 11, respectively.

The results of our analyses indicate that the biogeographic history of the sauropodomorphs included in this study is best explained in terms of sympatry, early occurrences of widespread ancestral stocks followed by regional extinction, and founder-event speciation. This also means that we find no compelling evidence for an important role for continent-scale vicariance. The lack of support for the latter might be a genuine reflection of the evolutionary history of these dinosaurs, but it is also conceivable that sampling biases, incorrect phylogenetic topology, and/or errors in the dating of cladogenetic and paleogeographic events, have disrupted any evidence for vicariance (e.g., see discussion in Upchurch [115] concerning the special requirements for detecting vicariance, and Poropat et al. [103] regarding interpretation of BioGeoBEARS results and sampling issues).

The ancestral area estimations for the relaxed and harsh BAYAREALIKE+J results are very similar, and are identical for the key selected nodes discussed below. According to these results, the most probable areas occupied by the most recent common ancestors (MRCAs) for the following clades are: Asia + South America (Neosauropoda, Diplodocoidea, Flagellicaudata, Dicraeosauridae, *Lingwulong*+advanced dicraeosaurids), and Asia + North America + South America (Macronaria). Such results are anomalous in the sense that there are no palaeogeographic reconstructions that support the existence of a single geographic unit that comprised Asia + South America, to the exclusion of other continental areas, during the Mesozoic. Following the reasoning of Poropat et al. [103], we suggest that these estimates indicate some, but not all, of the areas occupied by the ancestors. Potentially, the MRCAs of these various clades were widespread across Pangaea during the Middle Jurassic, occupying at least those areas that linked Asia and South America (such as Africa and Europe). We suggest that these other areas are not shown in the ancestral area estimations

because of sampling failures. This prediction can be tested in the future through additional discoveries of Jurassic sauropods in key areas such as the currently very poorly sampled Middle Jurassic of North America, sub-Saharan Africa, and mainland Europe, improved taxon sampling in phylogenetic analyses, and modifications to the BioGeoBEARS approach so that it can take into account data on spatial and temporal sampling heterogeneity.

6.6. Additional analysis and results

We re-ran our BioGeoBEARS analyses on a reduced dataset without *Tornieria*, *Dinheirosaurus*, *Supersaurus*, and *Leinkupal*. The time-calibrated tree used in this reduced analysis is presented in Supplementary Data 12, and the outputs for BAYAREALIKE and BAYAREALIKE+J for the relaxed and harsh analyses are presented in Supplementary Data 13 and 14, respectively. The results of both the relaxed and harsh analyses of this reduced dataset were very similar to those described above, with BAYAREALIKE+J being strongly preferred over other models (Supplementary Data 17). The relaxed analysis estimated that the ancestors of major clades such as Neosauropoda, Diplodocoidea, Flagellicaudata, and Macronaria were present in at least Asia, North America, and South America. Similarly, in the harsh analysis, the ancestral area estimation involves: Asia, North America, and South America for Neosauropoda, Diplodocoidea, and Flagellicaudata; Asia and South America for Dicraeosauridae; and Europe, North America, and South America for Macronaria (Supplementary Data 14). Thus, although the precise areas occupied by the various ancestral nodes differ in detail from those estimated by our full analysis (e.g., increased occupation of North America and the introduction of Europe as an ancestral area for Macronaria), our conclusions remain essentially unaltered. Both analyses suggest that neosauropods, and many of the major neosauropod subclades, were widespread across Pangaea during the late Early and/or early Middle Jurassic.

Supplementary Note 7: **Chinese sauropod biogeography, diversity, and sampling**

Despite the caveats above, our results support the hypothesis that neosauropods, including both macronarians and several diplodocoid lineages, were present in East Asia during the Middle and Late Jurassic, even though the only direct body fossil evidence for this comes from the early Middle Jurassic *Lingwulong* and possibly the Callovian/Oxfordian *Bellusaurus*. If correct, this result significantly undermines the EAIH and its corollaries relating to invasion by titanosauriforms during the Cretaceous in association with marine regressions [62, 63, 116, 117, 118, 119]. The EAIH and associated Cretaceous invasion hypothesis can be salvaged in a modified form [69] by proposing that: (1) although neosauropods reached East Asia during the Middle Jurassic, they did not manage to establish long-lasting lineages there (i.e., they died out before the start of the Cretaceous); (2) isolation during the Callovian–early Kimmeridgian produced endemism in East Asian dinosaurian faunas via vicariance; and (3) extinctions at or near the Jurassic/Cretaceous boundary, coupled with marine regressions, resulted in the replacement of endemic East Asian taxa such as mamenchisaurids

with titanosauriforms during the Early Cretaceous. However, this revised set of hypotheses represents a greatly diminished version of the EAIH, with isolation and endemism spanning just the late Middle and early Late Jurassic at most. As an alternative explanation for the currently available data, we propose the following linked hypotheses that emphasize a key role for uneven sampling of the fossil record:

(1) Neosauropods, and major constituent clades such as Macronaria and Diplodocoidea, originated in the early Middle Jurassic or late Early Jurassic – somewhat earlier than previously proposed (e.g., ref. [39]). At this time, Pangaea was still a coherent landmass, and neosauropods would have been able to disperse to all or most of its continental components, including East Asia.

(2) Diplodocoids and macronarians were present in East Asia during the Middle and Late Jurassic and into the Cretaceous (*Lingwulong*, *Bellusaurus*, the Thai diplodocoid), with additional waves of invasion by some advanced lineages, such as somphospondylan titanosauriforms, during the Cretaceous (perhaps still related to marine regressions).

(3) The scant representation of neosauropods in the Jurassic of East Asia results from uneven sampling, reflecting geologic and biotic factors such as uplift-driven erosion of key sedimentary basins and/or low abundance or habitat preferences (see below). Other factors have probably contributed to obscure the presence of East Asian neosauropods, including fragmentary preservation of putative neosauropods and poorly resolved phylogenetic placements caused by lack of study of key specimens (e.g., *Abrosaurus*).

The existence of rich sauropod faunas in the Middle and Late Jurassic of China, composed exclusively of non-neosauropods such as mamenchisaurids, has reinforced the view that the absence of neosauropods is unlikely to be a sampling artefact [62, 63]. However, despite the presence of a large amount of material (60 Chinese Middle and Late Jurassic collections containing sauropod specimens according to the Fossilworks Database [http://fossilworks.org/bridge.pl?a=home]), these are geographically clustered. In particular, there are major concentrations of sauropod-bearing units in Sichuan Province in southwest China (29 collections) and Xinjiang Autonomous Region in far western China (15 collections). With a further eight collections in Yunnan and Chongqing Provinces, this means that 52/60 (i.e., 86.7%) of these collections have come from the south or west of China. By contrast, apart from the new Lingwu locality reported in this study, the Ordos Basin in northwest China has yielded only four collections (three in Gansu and one in Shanxi) of Middle or Late Jurassic sauropod specimens. The rarity of Middle and Late Jurassic sauropods from the Ordos Basin could reflect: (1) low abundance and/or diversity of such taxa in the original ecosystems; (2) low preservation rates for large terrestrial vertebrates in the swamp- and lake-dominated Ordos Basin environments; (3) high rates of erosion subsequent to burial, resulting in large-scale destruction of the preserved fossils; and/or (4) a lack of prospecting/sampling effort by paleontologists. Factors 2 and 4 seem the least likely explanations for the lack of Jurassic Ordos specimens: the abundant fluviolacustrine burial environments do not seem to be qualitatively different from those elsewhere in China that have yielded significant quantities of dinosaur fossils, and the intensive prospecting of the Ordos Basin for coal, oil, uranium, and other economically important resources (e.g., refs. [4, 9, 13]) means that these deposits have been

scrutinized by geologists and palaeontologists who would have reported dinosaur specimens if they were present. In contrast, there is evidence for several episodes of erosion related to uplift of the Ordos Basin during the Middle and early Late Jurassic, manifested in the disconformities between several key formations (Yanan, Zhiluo, and Anding) (e.g., refs. [5, 8, 10, 12, 22, 23]). Thus, some dinosaur-bearing deposits are likely to have been lost during the uplift and erosion of the Ordos Basin. While this factor might have reduced the absolute quantity of preserved dinosaur material, it remains the case that the Yanan, Zhiluo, and Anding formations have yielded numerous invertebrate and plant macrofossils, as well as abundant microfossils, and substantial thicknesses of sediments have been explored for both their fossil and mineral content: the scarcity of large tetrapod remains might therefore reflect genuinely low abundance and/or diversity of such taxa in northwest China during the Middle Jurassic. Moreover, the absence of neosauropods in the well-sampled southwest of China (Sichuan) might indicate that members of this clade were geographically restricted to northern and perhaps western regions (partly depending on whether *Bellusaurus* from Xinjiang is a macronarian). Interestingly, the sole North American dicraeosaurid, *Suuwassea*, has, so far, only been found in the northern part of the Morrison Formation (Montana), at a palaeolatitude of ~41° N, despite the discovery of abundant and well-preserved sauropod specimens further south (e.g., ref. [54]; Fossilworks Database). Lingwu is estimated to have been at a palaeolatitude of ~44° N at 175 Ma, and other dicraeosaurids have been found at sites in the ranges of 38–41° palaeolatitude from the equator, apart from *Dicraeosaurus* at ~30–32° S (Fossilworks Database). This could potentially indicate some latitudinal control on dicraeosaurid geographic ranges, perhaps mediated by the effects of climate on habitat distributions, but this hypothesis is difficult to test at present given the current rarity of members of this clade.

Supplementary Figures

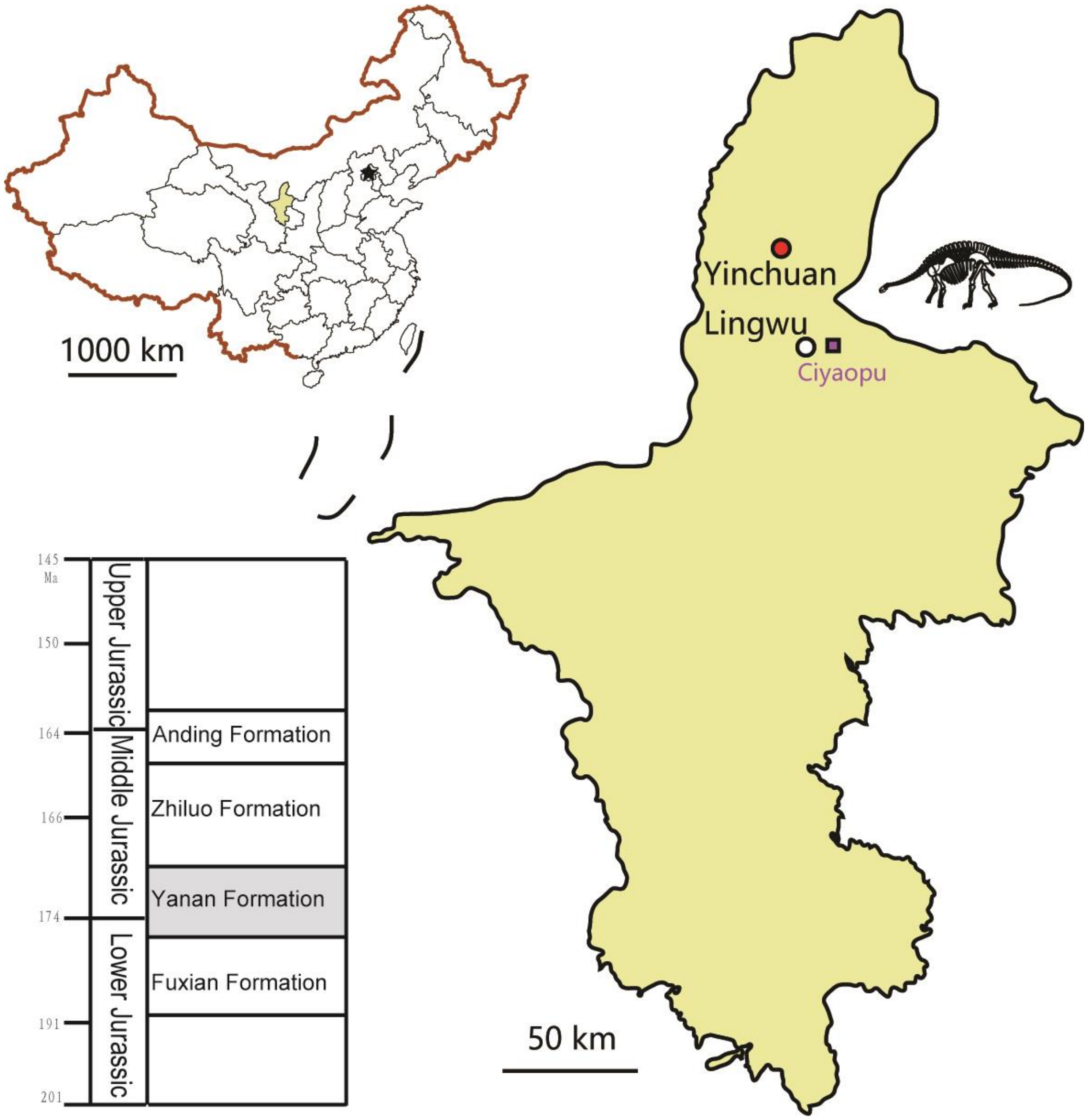

Supplementary Fig. 1. Geographic location and stratigraphy of the Ciyaopu sauropod quarry. Map showing Ningxia Hui Autonomous Region (shaded yellow) within China (upper left), with magnified inset showing Lingwu and Ciyaopu within Ningxia (right). The stratigraphic setting of the fossil-bearing Yanan Formation and its relationships to other Jurassic formations in Ningxia is shown lower left.

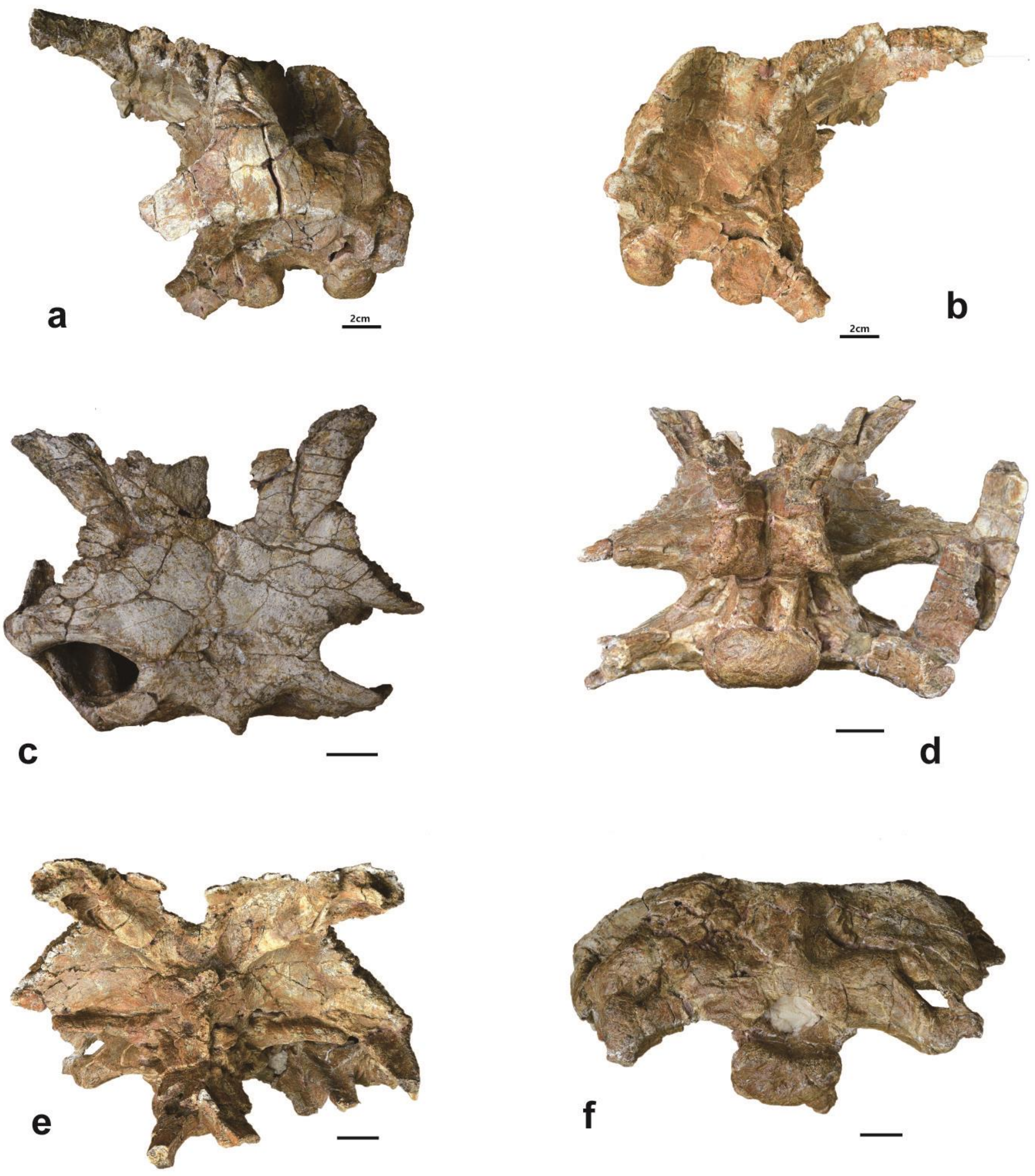

Supplementary Fig. 2. Braincase of *Lingwulong*. Braincase in left (a) and right (b) lateral views, dorsal (c), ventral (d), anterior (e), and (f) occipital views. Scale bar = 2 cm.

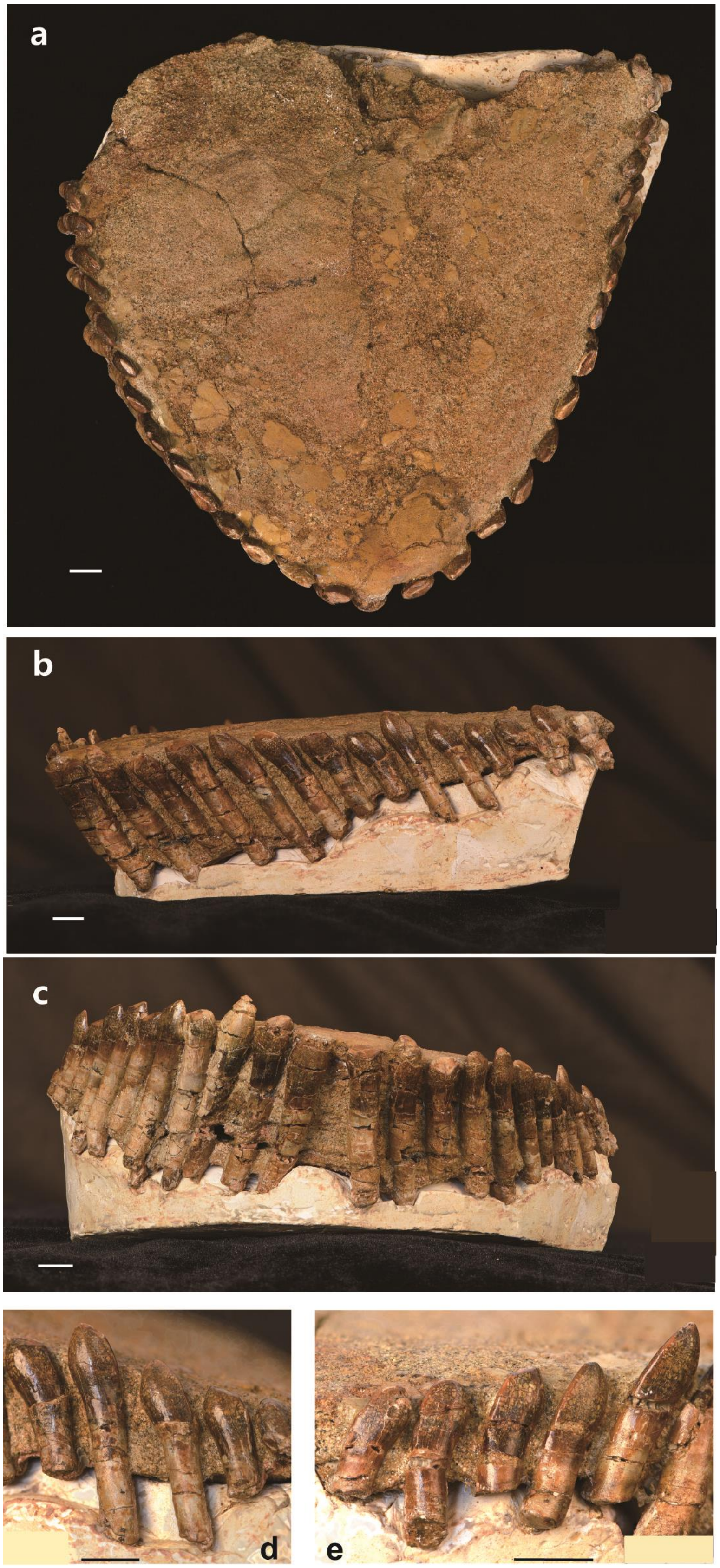

Supplementary Fig. 3. Dentary tooth row of *Lingwulong*. Twenty-nine preserved dentary teeth in apical (a), left labial (b), and anterior (c) views; close-ups of selected right (d) and left (e) dentary teeth in labial views. Scale bar = 1 cm.

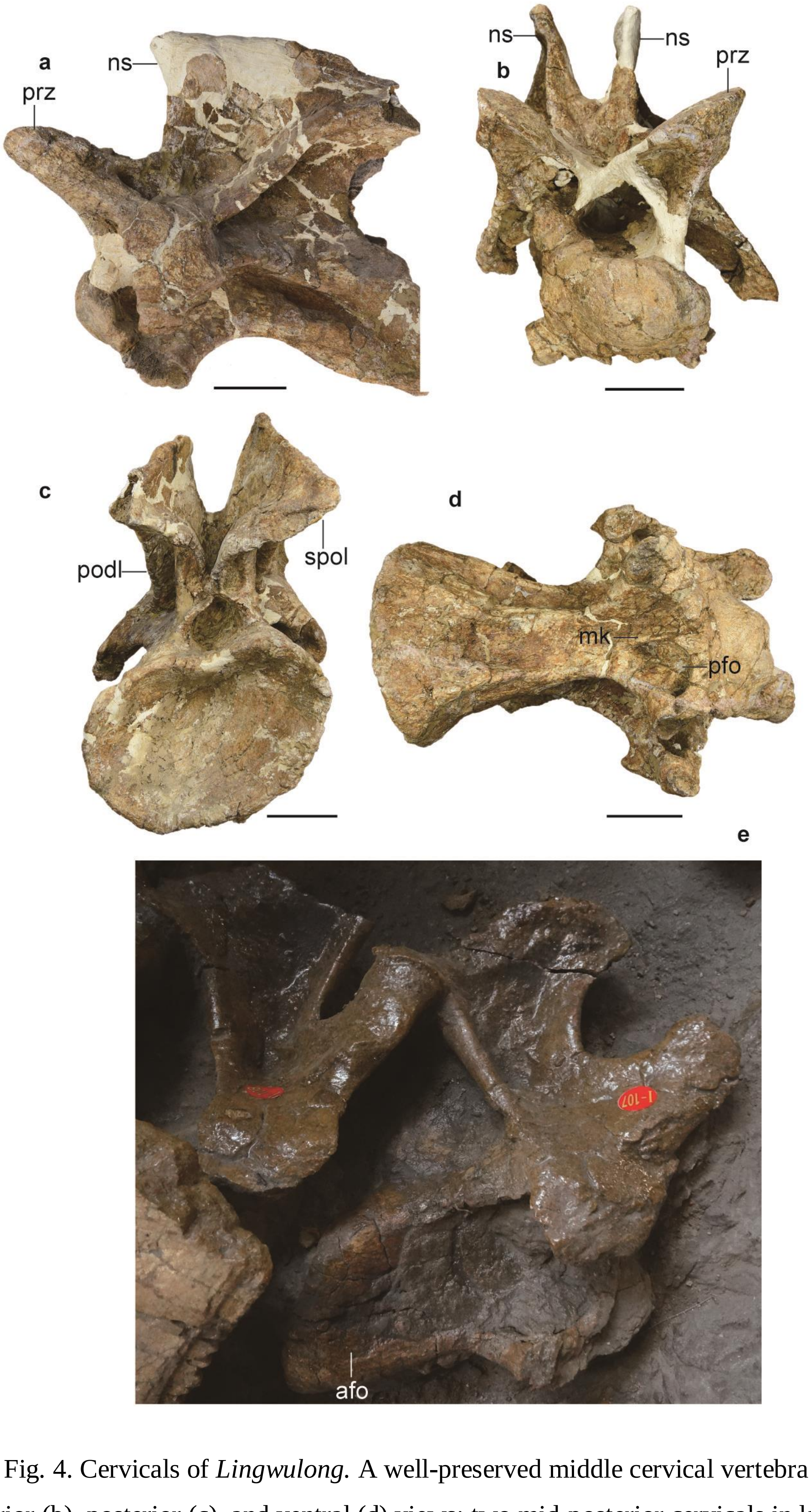

Supplementary Fig. 4. Cervicals of *Lingwulong.* A well-preserved middle cervical vertebra in lateral (a), anterior (b), posterior (c), and ventral (d) views; two mid-posterior cervicals in lateral view (e). Abbreviations: afo, accessory fossa; mk, midline keel; np, neural spine; pf, pneumatic fossa; podl, postzygodiapophyseal lamina; prz, prezygapophysis; spol, spinopostzygapophyseal lamina. Scale bar = 5 cm.

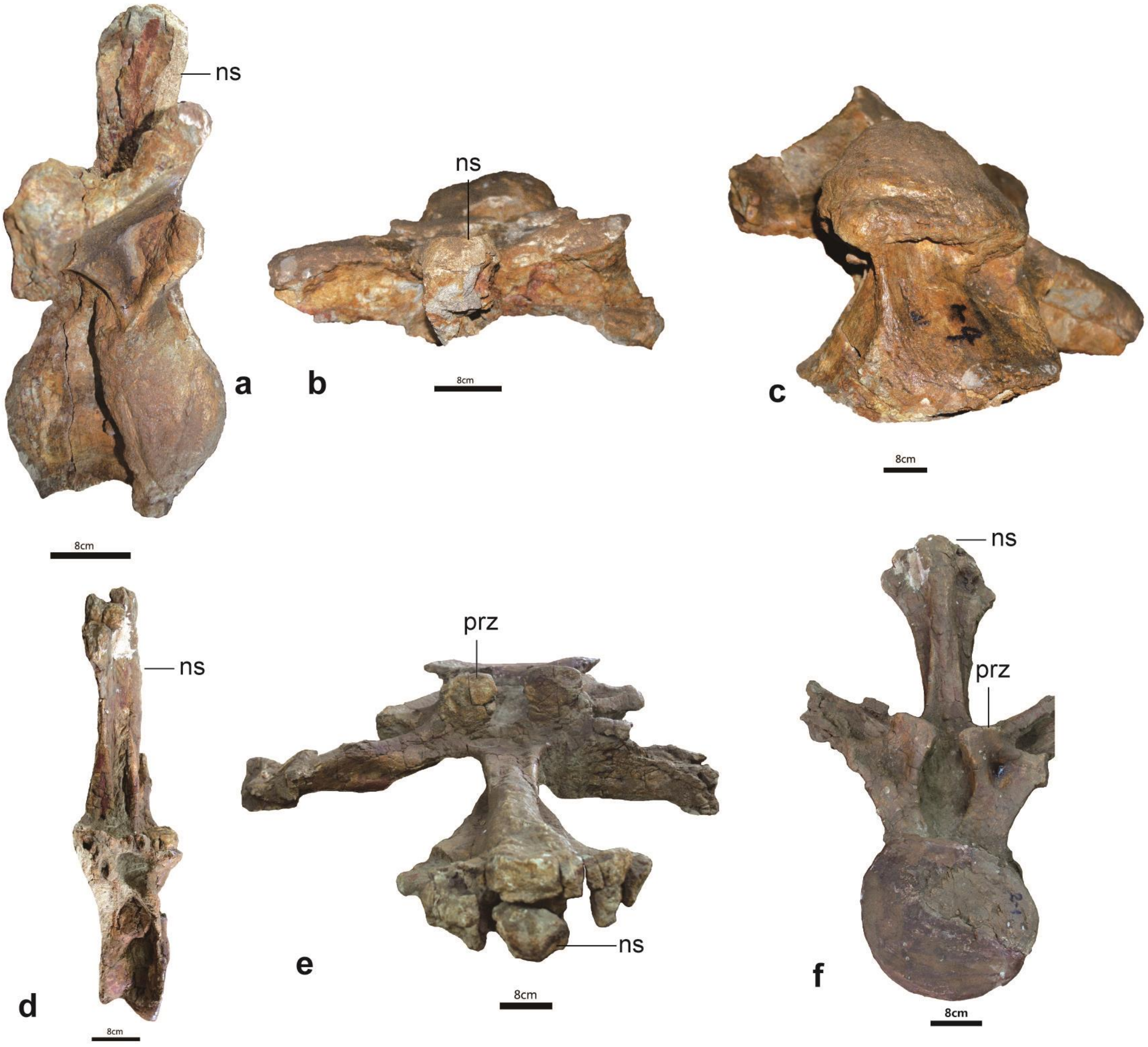

Supplementary Fig. 5. Anterior and middle dorsal vertebrae of *Lingwulong*. An anterior dorsal vertebra in lateral (a), dorsal (b), and ventral (c) views; a middle dorsal vertebra in lateral (d), dorsal (e), and anterior (f) views. Abbreviations: ns, neural spine; prz, prezygapophysis. Scale bar = 8 cm.

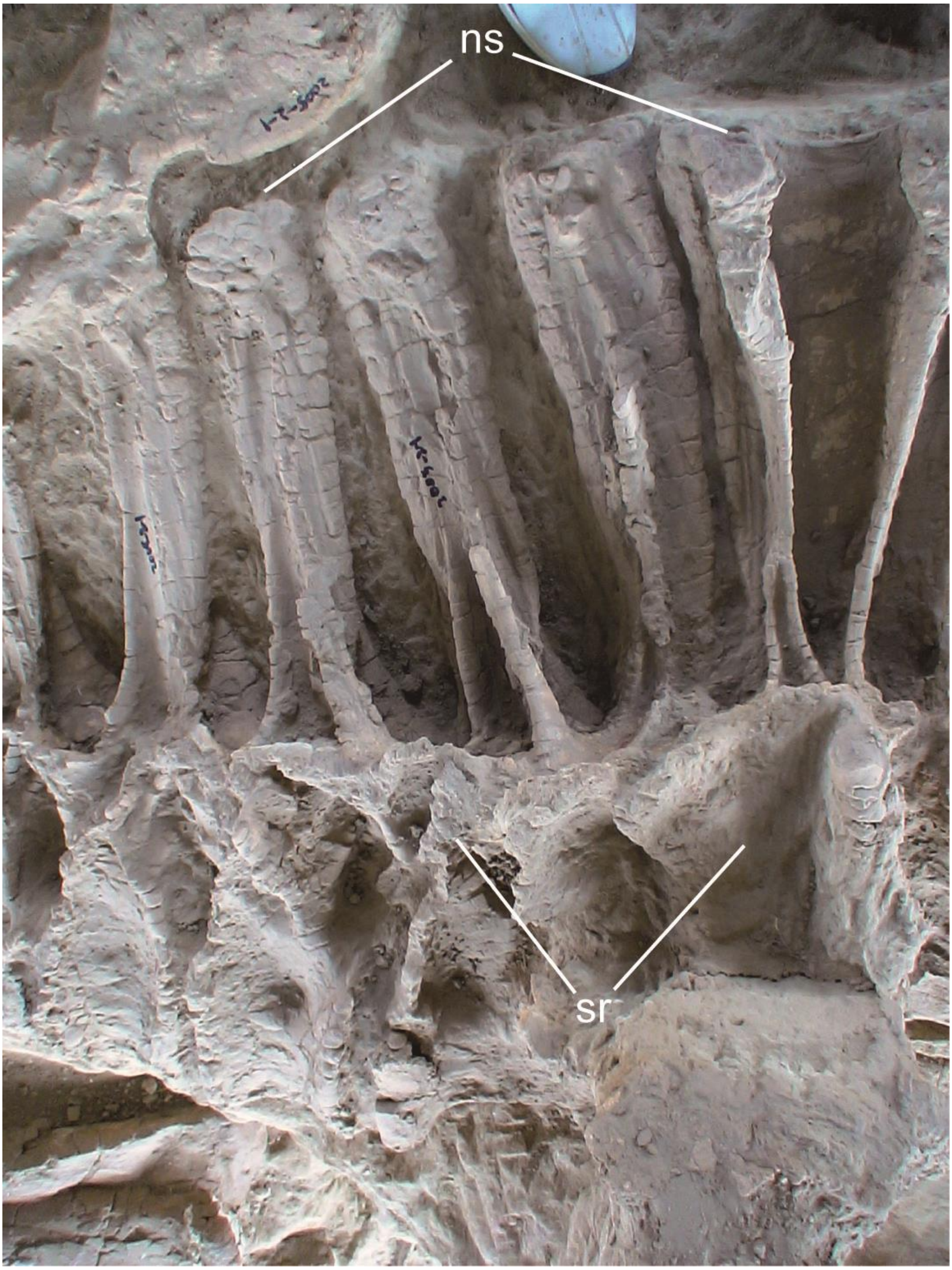

Supplementary Fig. 6. Sacrum of *Lingwulong* in lateral view, showing spine fusion and laminae. Abbreviations: ns, neural spine; sr, sacral rib.

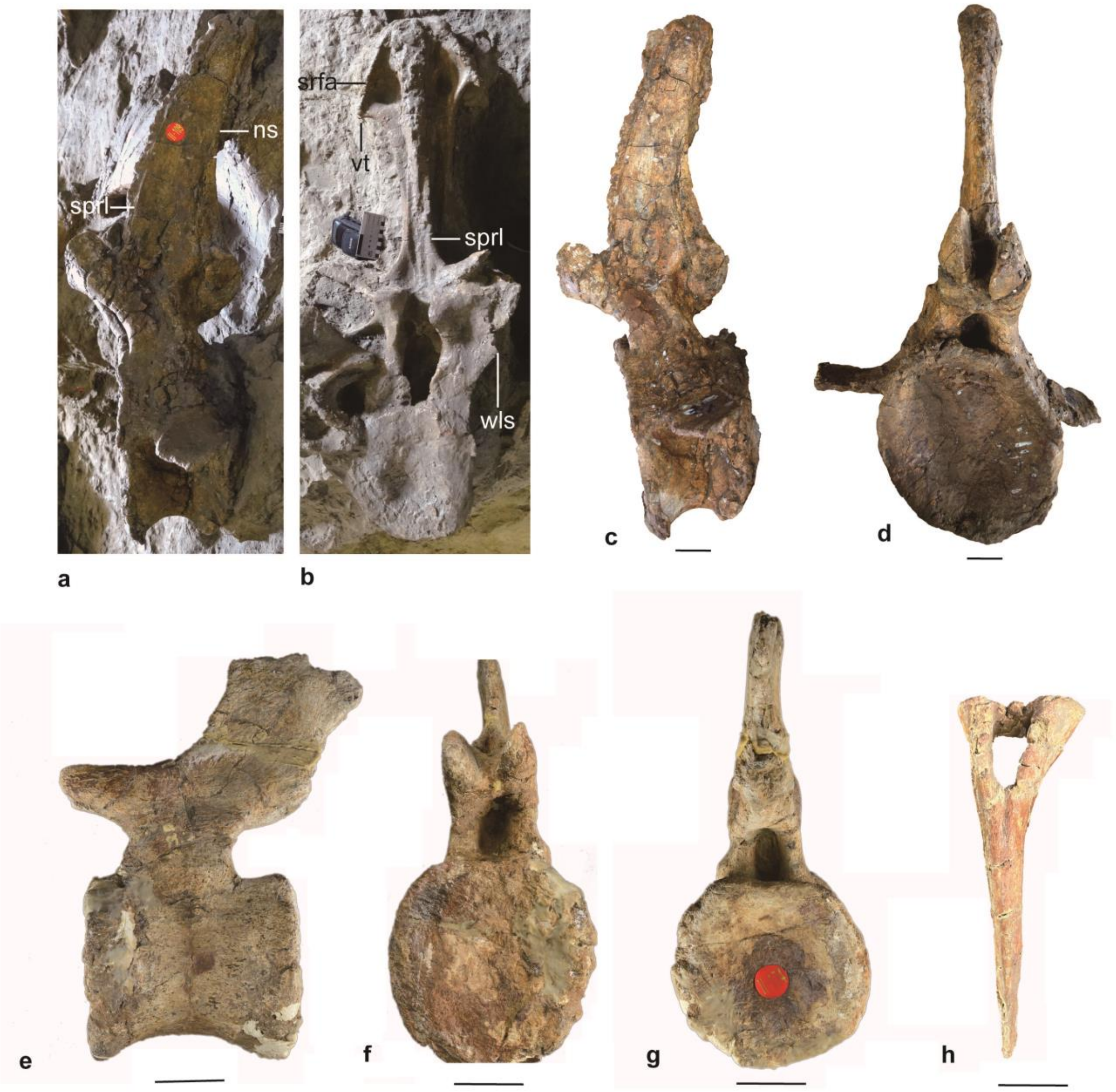

Supplementary Fig. 7. Caudals and chevron of *Lingwulong*. An anterior-most caudal vertebra in lateral (a) and anterior (b) views; an anterior caudal vertebra in lateral (c) and anterior (d) views; a middle caudal vertebra in lateral (e), anterior (f), and posterior (g) views; an anterior chevron in anterior view (H). Abbreviations: ns, neural spine; sprl, spinoprezygapophyseal lamina; stfa, subtriangular facet-like area; vt, ventral tip; wls, wing-like structure. Scale bar = 5 cm.

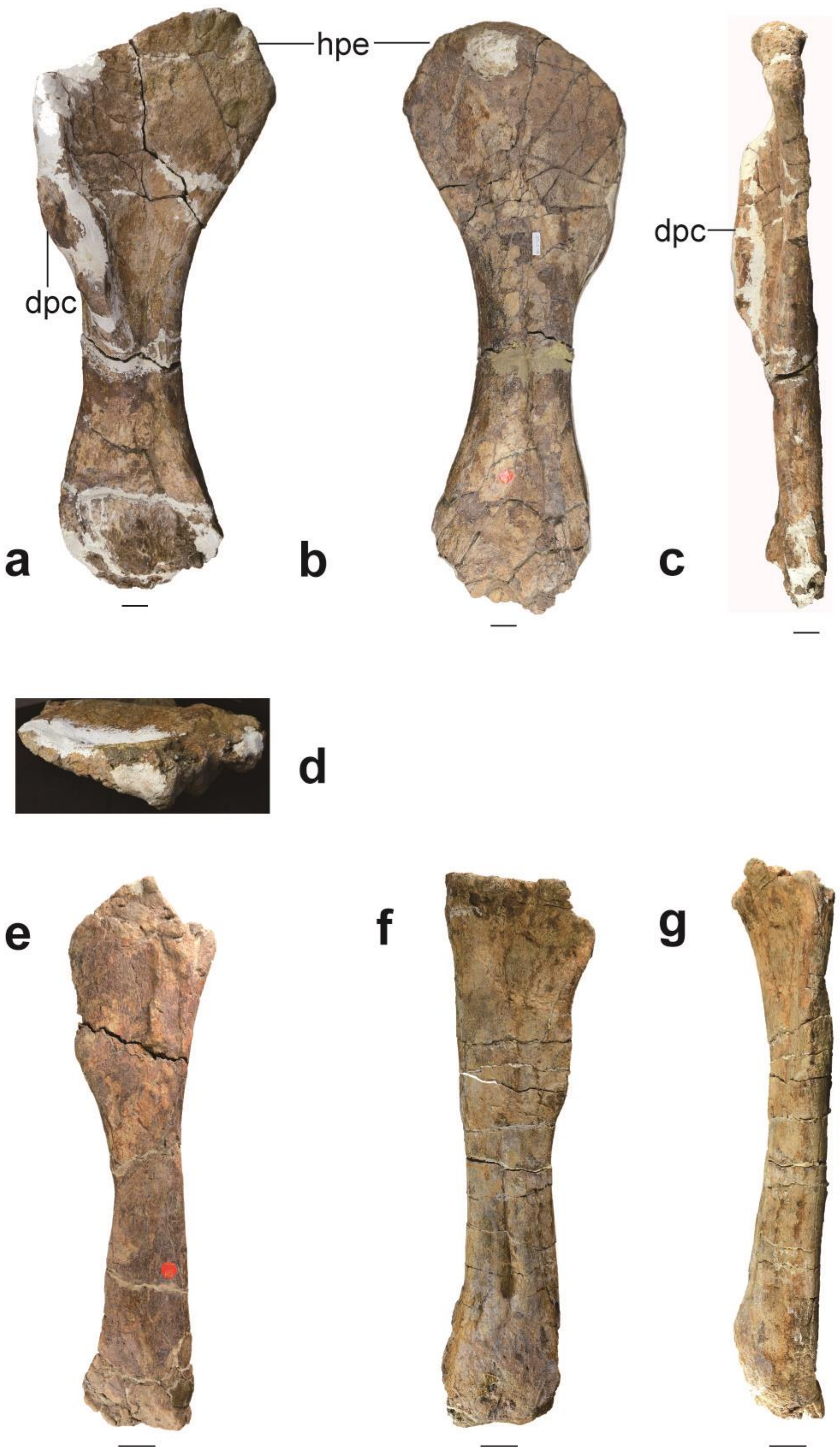

Supplementary Fig. 8. Right humerus, ulna, and radius of *Lingwulong*. Right humerus in anterior (a), posterior (b), and lateral (c) views; right ulna in proximal (d) and lateral (e) views; right radius in anterior (f) and lateral (g) views. Abbreviations: dpc, deltopectral crest; hpe, humeral proximal end. Scale bar = 5 cm.

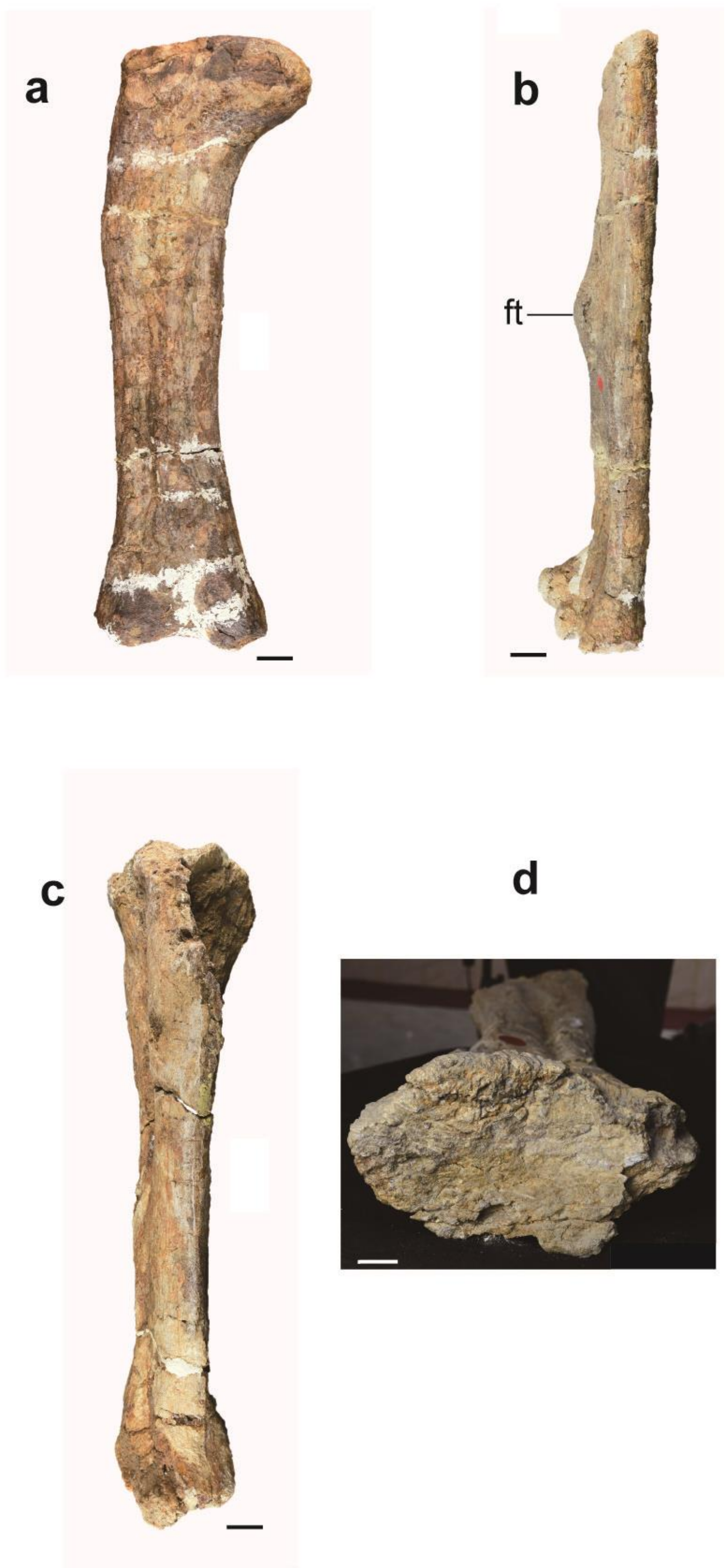

Supplementary Fig. 9. Right femur and left tibia of *Lingwulong*. Right femur in anterior (a) and lateral (b) views; left tibia in lateral (c) and proximal (d) views. Abbreviations: ft, fourth trochanter. Scale bars = 5 cm for (a–c) and 2 cm for (d).

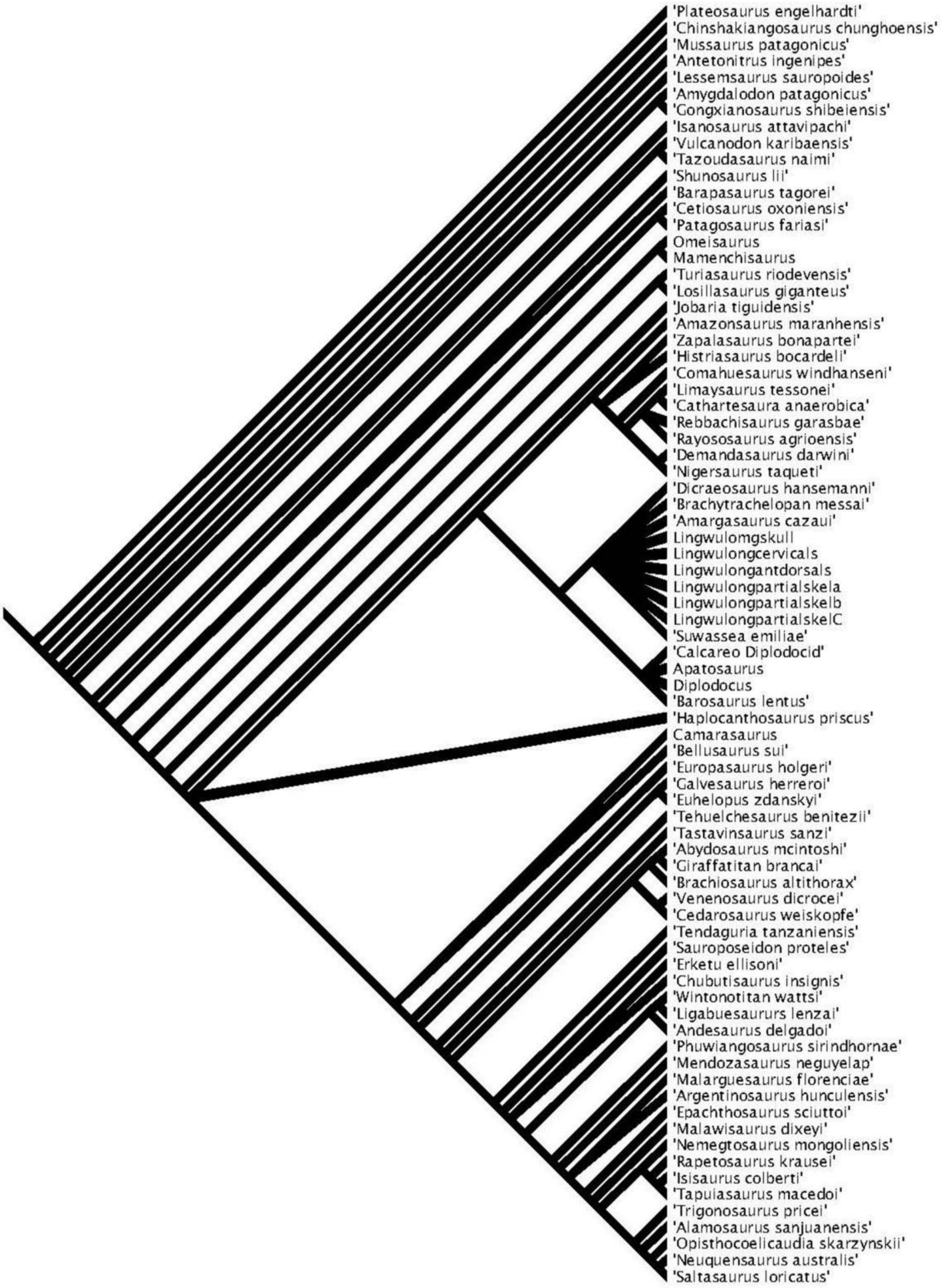

Supplementary Fig. 10. Main dataset. Strict consensus tree showing the relationships of the six specimen-level OTUs based on sauropod material from the Lingwu Geopark locality (see Supplementary Note 4 for details).

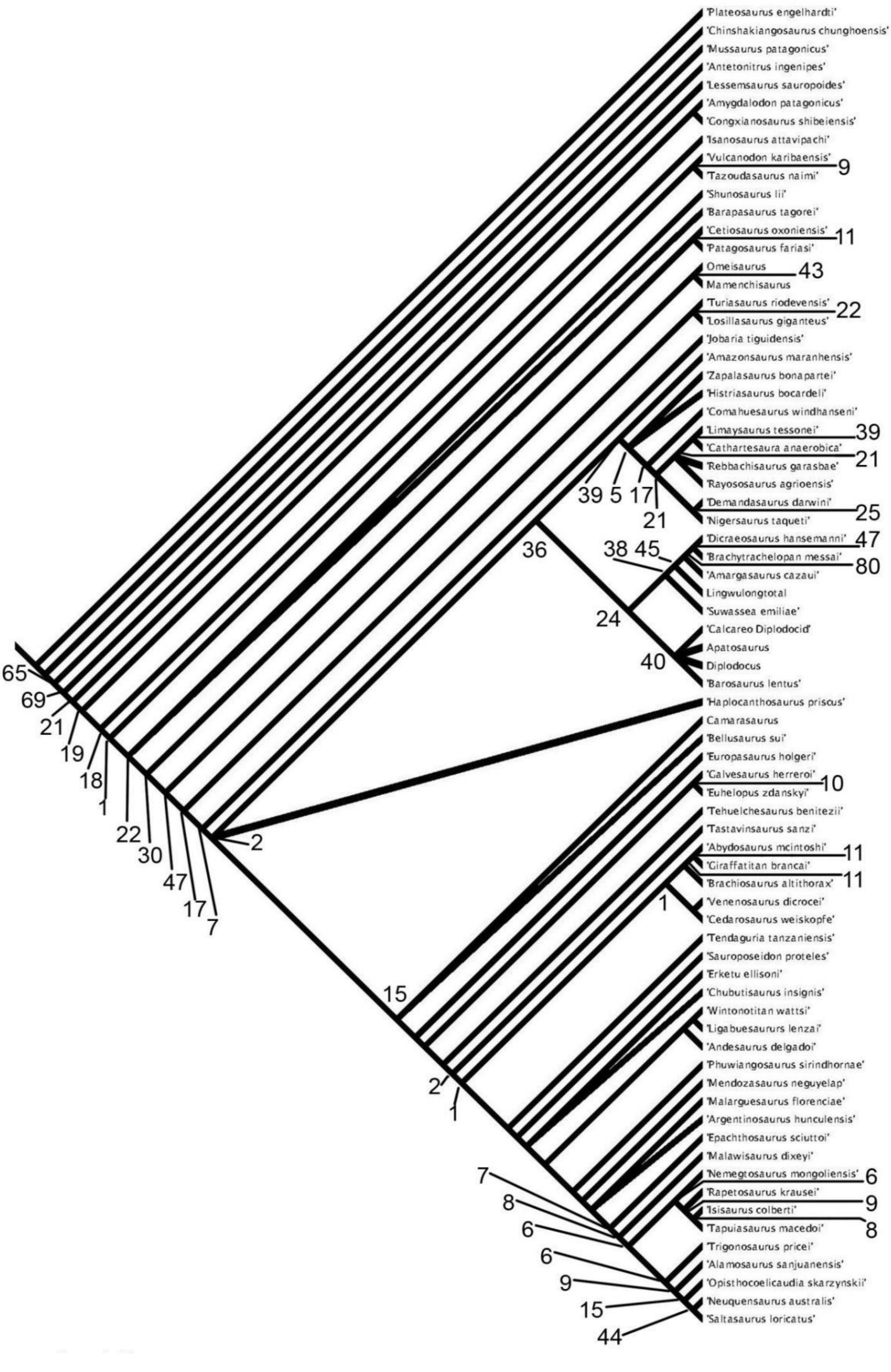

Supplementary Fig. 11. Main dataset. Strict consensus tree showing the relationships of the composite OTU 'Lingwulongtotal'. GC values (multiplied by 100) are shown as numbers at nodes (N.B., nodes with 0 or

minus GC values are left blank). The summary topology generated by the symmetric resampling analyses is slightly different from the strict consensus tree shown here: this discrepancy is interpreted to have arisen as a result of the use of a TBR search in the symmetric resampling as opposed to a New Technology Search plus TBR search when generating the MPTs, and/or the effects of randomly re-weighting characters during the symmetric resampling analyses. The symmetric resampling topology has the following relationships that are not shown in the strict consensus tree: (1) *Amygdalodon* is more closely related to eusauropods than is *Gongxianosaurus*; (2) *Barapasaurus* is more closely related to the clade defined by *Cetiosaurus*+*Saltasaurus* than is *Shunosaurus*; (3) *Haplocanthosaurus* is resolved as the sister-taxon to other diplodocoids; (4) *Camarasaurus* and *Bellusaurus* are sister taxa (GC = 15); and (5) *Venenosaurus* is more closely related to the *Brachiosaurus*+(*Giraffatitan*+*Abydosaurus*) clade than to *Cedarosaurus*.

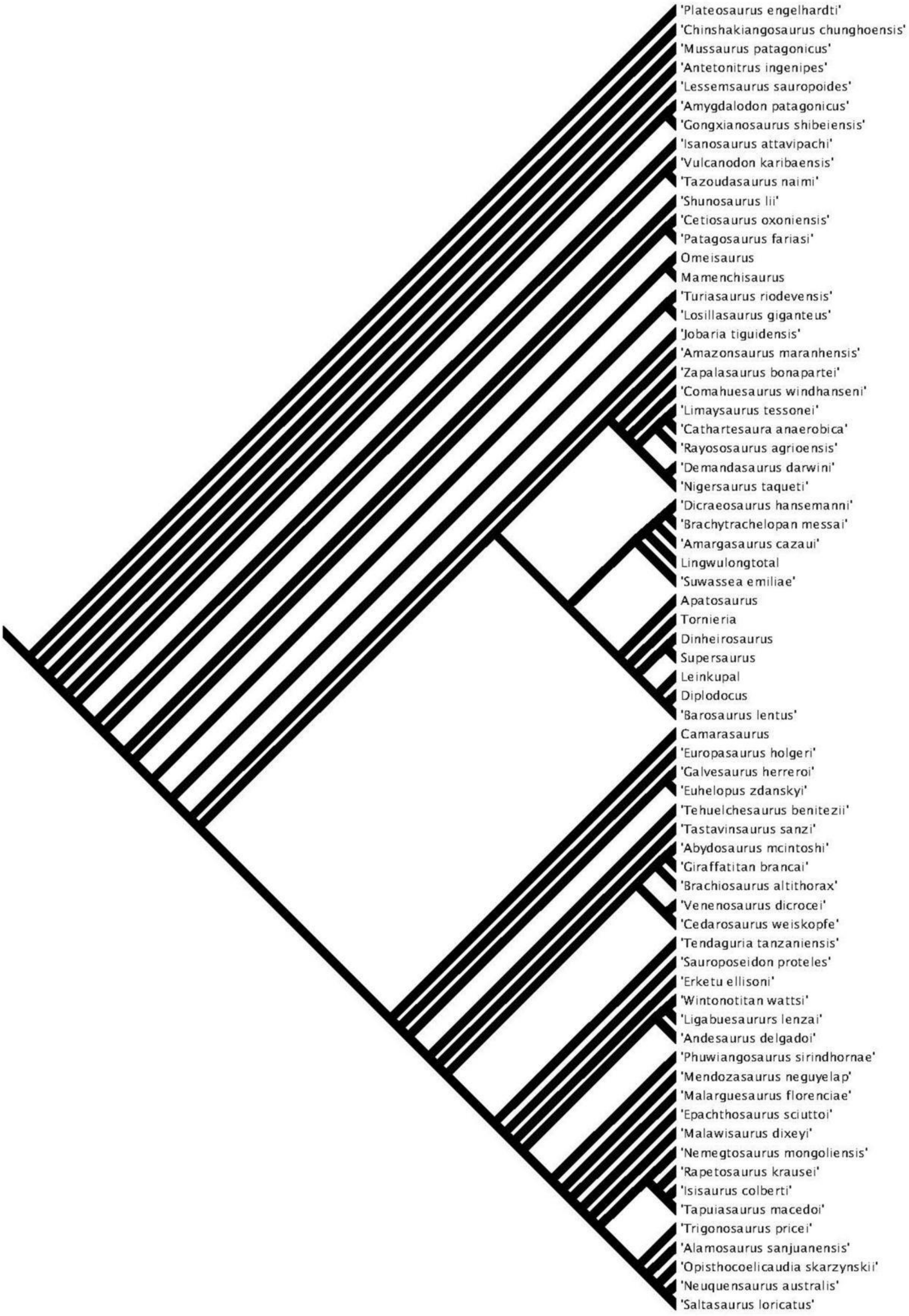

Supplementary Fig. 12. Main dataset. The agreement subtree generated in TNT via the a posteriori deletion of eight OTUs from the 12 MPTs. Four diplodocid taxa (*Dinheirosaurus*, *Leinkupal*, *Supersaurus*, and *Tornieria*) have been manually grafted onto this tree based on their relationships in Tschopp et al. [27] (see Supplementary Note 6 for justification of these additions).

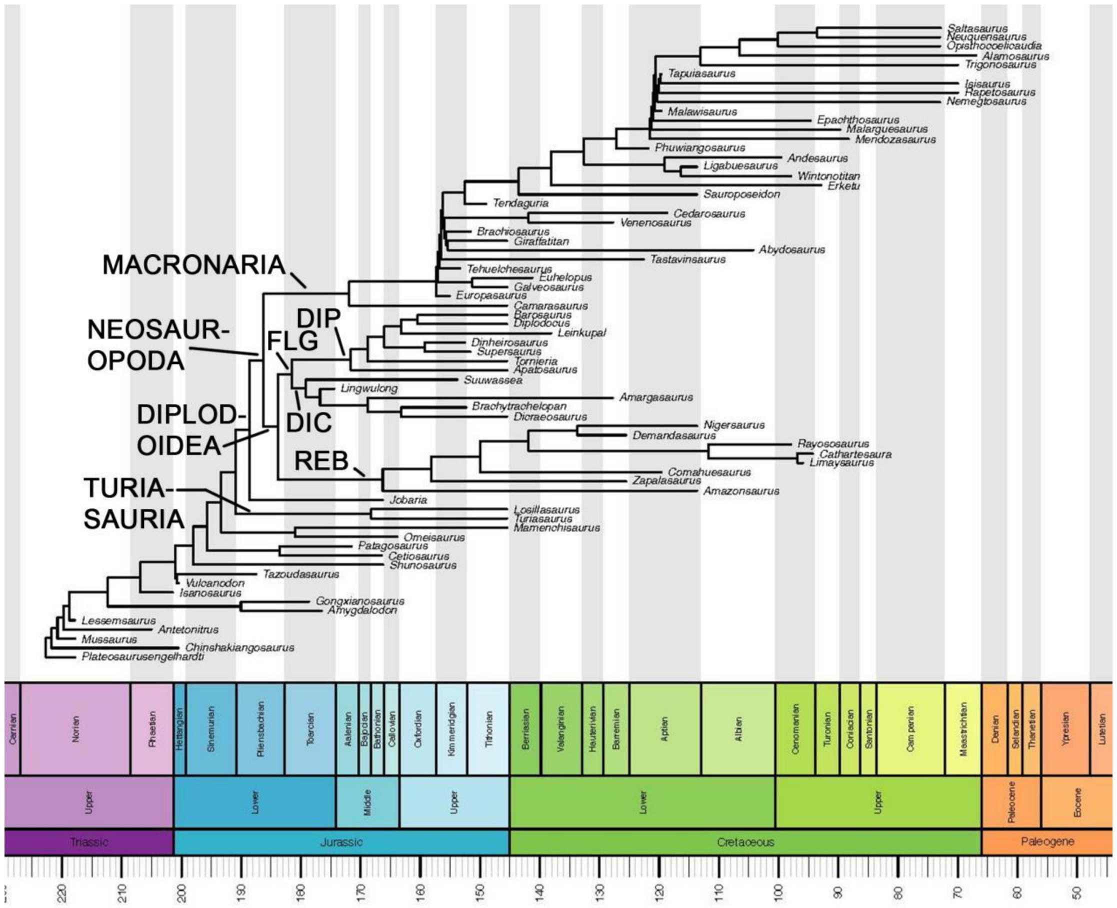

Supplementary Fig. 13. Main dataset. The time-calibrated phylogeny for sauropods based on our augmented agreement subtree (Supplementary Fig. 12) and the taxon ages given in Supplementary Data 3. This was created using the R package strap [99]. Abbreviations: DIC, Dicraeosauridae; DIP, Diplodocidae; FLG, Flagellicaudata; REB, Rebbachisauridae. See also Fig. 3.

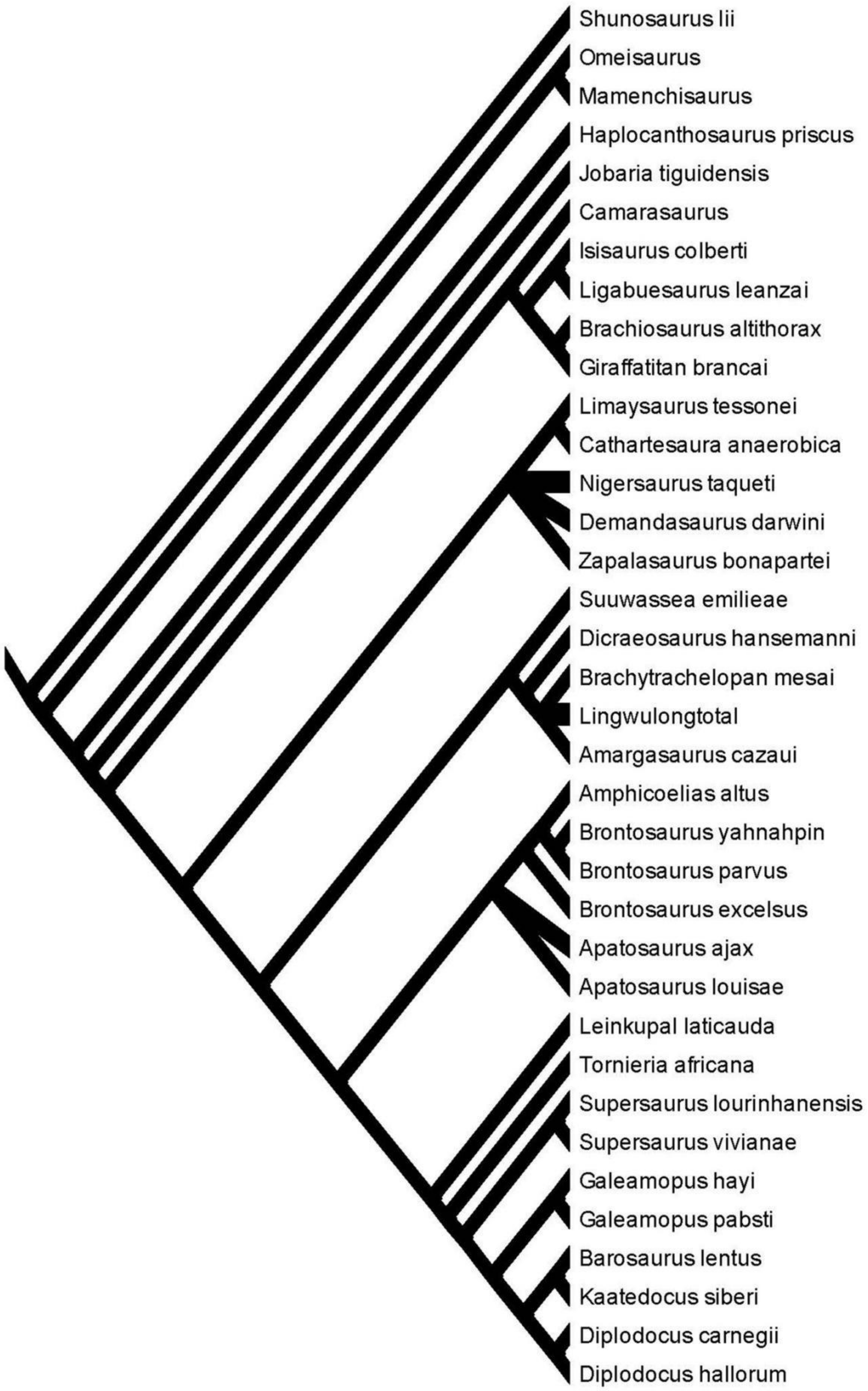

Supplementary Fig. 14. Subsidiary dataset. Strict consensus tree (based on the 12 MPTs produced by equal weights parsimony) showing the relationships of the composite OTU 'Lingwulongtotal'.

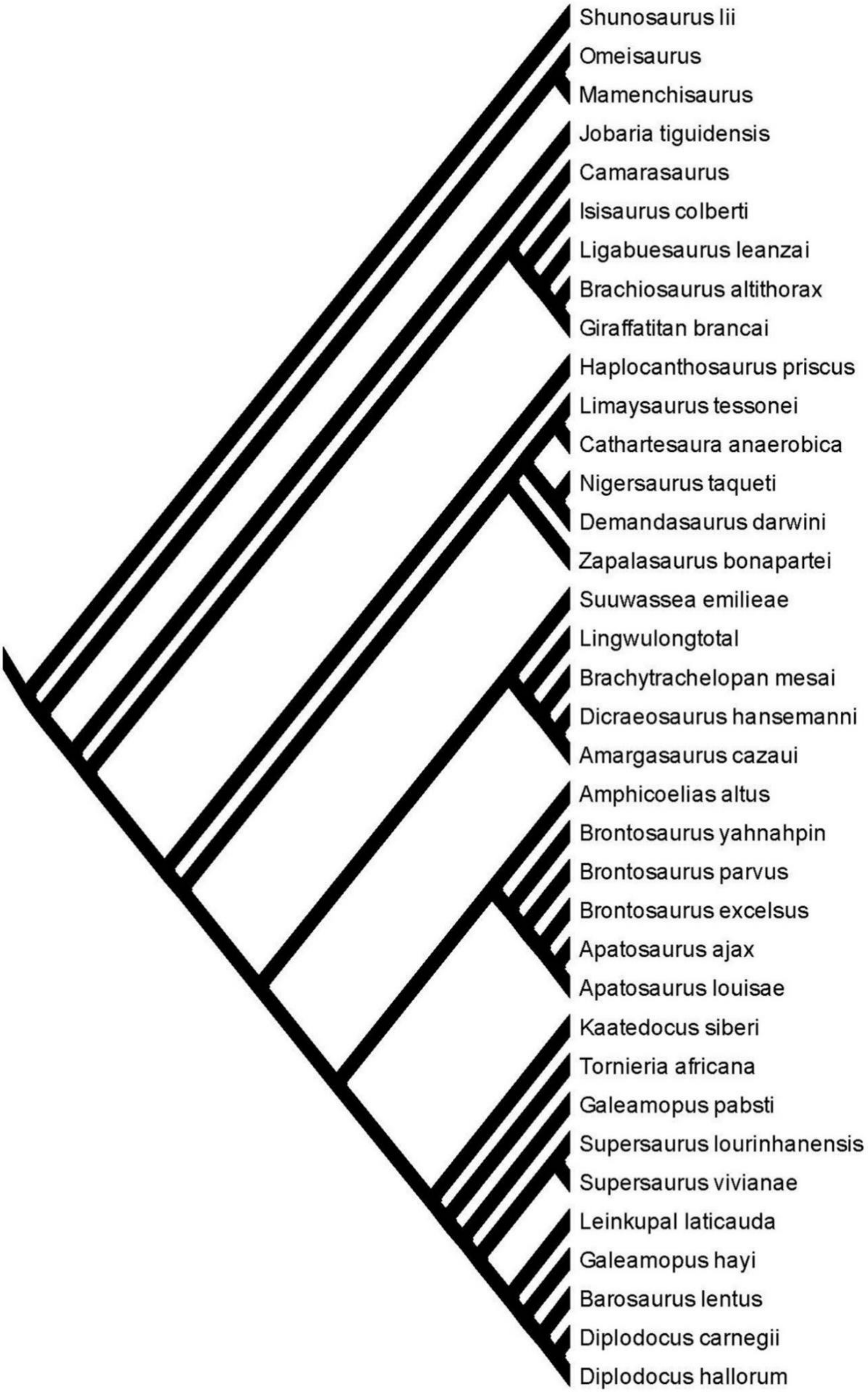

Supplementary Fig. 15. Subsidiary dataset. The single MPT produced by both basic and Extended Implied Weighting, showing the relationships of the composite OTU 'Lingwulongtotal'.

Supplementary References

1. Wilson JA. A nomenclature for vertebral laminae in sauropods and other saurischian dinosaurs. *Journal of Vertebrate Paleontology* **19**, 639-653 (1999).

2. Wilson JA, D'Emic MD, Ikejiri T, Moacdieh EM, Whitlock JA. A nomenclature for vertebral fossae in sauropods and other saurischian dinosaurs. *PLoS ONE* **6**, e17114 (2011).

3. Kimura T. Mesozoic Floras of East and Southeast Asia, with a Short Note on the Cenozoic Floras of Southeast Asia and China. *Geol Palaeontology of Southeast Asia* **25**, 325–350 (1984).

4. Johnson EA, Liu S, Zhang Y. Depositional environments and tectonic controls on the coal-bearing lower to Middle Jurassic Yan'an Formation, southern Ordos Basin, China. *Geology* **17**, 1123–1126 (1989).

5. Chen J. Evolution of palaeogeography during the Mesozoic. In: *Phanerozoic Geology of Northwest China* (eds Zhou Z, Dean WT) 115–123 (Science Press, 1996).

6. Li D, Dong S, Deng S. New knowledge of the Upper Triassic in Liupanshan Basin, Ningxia, China. *Chinese Science Bulletin* **43**, 1100–1107 (1998).

7. Chen P-J, Li J, Matsukawa M, Zhang H, Wang Q, Lockley MG. Geological ages of dinosaur-trackbearing formations in China. *Cretaceous Research* **27**, 22–32 (2006).

8. Wang Y, Dong S, Zhao Y, Zhang T. Jurassic Tectonics of North China: A Synthetic View. *Acta Geological Sinica (English edition)* **82**, 310–326 (2008).

9. Li X, *et al.* Hydrocarbon origin and reservoir forming model of the Lower Yanchang Formation, Ordos Basin. *Petroleum Exploration and Development* **39**, 184–193 (2012).

10. Tanner LH, Wang X, Morabito AC. Fossil charcoal from the Middle Jurassic of the Ordos Basin, China and its paleoatmospheric implications. *Geoscience Frontiers Volume* **3**, 493–502 (2012).

11. Xing L, *et al.* Theropod and ornithischian footprints from the Middle Jurassic Yanan Formation of Zizhou County, Shaanxi, China. *Ichnos* **22**, 1–11 (2015).

12. Chen J. Mesozoic. In: *Phanerozoic Geology of Northwest China* (eds Zhou Z, Dean WT) 252–273 (Science Press, 1996).

13. Deng S, Fang L, Lu Y, Fan R, Yuan X. Field Guide: The Mesozoic Stratigraphy of the Ordos Basin. In: *The 8th International Congress on the Jurassic System* (eds) (2010).

14. Li G, *et al.* Confirmation of a Middle Jurassic age for the Eedemt Formation in Dundgobi Province, southeast Mongolia: constraints from the discovery of new spinicaudatans (clam shrimps). *Alcheringa* **38**, 305–316 (2014).

15. Li G, Matsuoka A. Jurassic clam shrimp ("conchostracan") faunas in China. *Science Report of Niigata University (Geology)* **27**, 73–88 (2012).

16. Zhao Y, Cui S, Guo T, Xu G. Evolution of a Jurassic basin of the Western Hills, Beijing, North China and its tectonic implications. *Geol Bull China* **21**, 211–217 (2002).

17. Zhao Y, Song B, Zhang S, Liu J. Geochronology of inherited zircons from Jurassic Nadaling basalt of Western Hills of Beijing, North China: its implication. *Earth Sci Frontier* **13**, 184–190 (2006).

18. Xu G, Zhao Y, Wu T, Zhang S. Late Triassic-Middle Jurassic stratigraphic succession in the Niuyingzi basin, Lingyum County, western Liaoning and the correlation of regional stratigraphic sequences in the Yanliao region. *Geosci Sinica* **26**, 299-308 (2005).

19. Chen J, Yang H. Geological development of the northwest China basins during the Mesozoic. In: *Phanerozoic Geology of Northwest China* (eds Zhou Z, Dean WT) 39–64 (Science Press, 1996).

20. Wu S, Tong J. Jurassic. In: *The Palaeobiogeography of China* (eds Yin H) 219–245 (Clarendon Press, 1994).

21. Zhou Z. Jurassic floras. In: *Fossil floras of China through the geological ages* (eds Li X, Zhou Z, Cai C, Sun G, Ouyang S, Deng L) 343–410 (Guangdong Science and Technology Press, 1995).

22. Zhang Y, Liao C, Shi W, Zhang T, Guo F. Jurassic deformation in and around the Ordos Basin, North China. *Earth Science Frontiers* **14**, 182–196 (2007).

23. Li S-H, Xi S-L, Feng S-B, Liu X-S. 150 Million year history of North China Craton disruption preserved in Mesozoic sediments of the Ordos basin. *International Geology Review* **58**, 1417–1442 (2016).

24. International Commission on Stratigraphy Chronostratigraphic Chart 2016 (see http://www.stratigraphy.org/index.php/ics-chart-timescale

25. Whitlock J. A phylogenetic analysis of Diplodocoidea (Saurischia: Sauropoda). *Zoological Journal of the Linnean Society* **161**, 872-915 (2011).

26. Mannion PD, Upchurch P, Mateus O, Barnes RN, Jones MEH. New information on the anatomy and systematic position of *Dinheirosaurus lourinhanensis* (Sauropoda: Diplodocoidea) from the Late Jurassic of Portugal, with a review of European diplodocoids. *Journal Systematic Palaeontology* **10**, 521–551 (2012).

27. Tschopp E, Mateus O, Benson RBJ. A specimen-level phylogenetic analysis and taxonomic revision of Diplodocidae (Dinosauria, Sauropoda). *PeerJ* **3**, e857 (2015).

28. Upchurch P, Barrett PM, Dodson P. Sauropoda. In: *The Dinosauria (second edition)* (eds Weishampel DB, Dodson P, Osmolska H) 259-322 (University of California Press, 2004).

29. Wilson JA. Sauropod dinosaur phylogeny: critique and cladistic analysis. *Zoological Journal of the Linnean Society* **136**, 217-276 (2002).

30. Harris JD. Cranial osteology of *Suuwassea emilieae* (Sauropoda: Diplodocoidea: Flagellicaudata) from the Upper Jurassic Morrison Formation of Montana, USA. *Journal of Vertebrate Paleontology* **26**, 88-102 (2006).

31. Mannion PD, Upchurch P, Barnes RN, Mateus O. Osteology of the Late Jurassic Portuguese sauropod dinosaur *Lusotitan atalaiensis* (Macronaria) and the evolutionary history of basal titanosauriforms. *Zoological Journal of the Linnean Society* **168**, 98–206 (2013).

32. Janensch W. *Palaeontographica (Supplement VII)* **2**, 147–298 (1935-36).

33. Whitlock J. Inferences of diplodocoid (Sauropoda: Dinosauria) feeding behavior from snout shape and microwear analyses. *PLoS ONE* **6**, e18304 (2011).

34. Janensch W. Die Wirbelsaule der Gatttung *Dicraeosaurus*. *Palaeontographica, Supplement* **7(1)**, 2(1): 37-133 (1929).

35. Rauhut OWM, Remes K, Fechner R, Cladera G, Puerta P. Discovery of a short-necked sauropod dinosaur from the Late Jurassic period of Patagonia. *435*, 670–672 (2005).

36. Cerda IA, Salgado L, Powell JE. Extreme postcranial pneumaticity in sauropod dinosaurs from South America. *Paläontologische Zeitschrift* **86**, 441–449 (2012).

37. Otero A, Gallina PA, Canale JI, Haluza A. Sauropod haemal arches: morphotypes, new classification and phylogenetic aspects. *Historical Biology* **24**, 243–256 (2012).

38. Mocho P, Royo-Torres R, Ortega F. Phylogenetic reassessment of *Lourinhasaurus alenquerensis*, a basal Macronaria (Sauropoda) from the Upper Jurassic of Portugal. *Zoological Journal of the Linnean Society* **170**, 87–916 (2014).

39. Rauhut OWM, L. CJ, Pol，Diego. A diplodocid sauropod dinosaur from the Late Jurassic Cañadón Calcáreo Formation of Chubut, Argentina. *Journal of Vertebrate Paleontology* **35**, (2015).

40. Carballido JL, Sander PM. Postcranial axial skeleton of Europasaurus holgeri (Dinosauria, Sauropoda) from the Upper Jurassic of Germany: implications for sauropod ontogeny and phylogenetic relationships of basal Macronaria. *Journal of Systematic Palaeontology* **12**, 335–387 (2014).

41. Carballido JL, Rauhut OWM, Pol D, Salgado L. Osteology and phylogenetic relationships of *Tehuelchesaurus benitezii* (Dinosauria, Sauropoda) from the Upper Jurassic of Patagonia. *Zoological Journal of the Linnean Society* **163**, 605–662 (2011).

42. Carballido J, Pol D, Cerda I, Salgado L. The osteology of Chubutisaurus insignis del Corro, 1975 (Dinosauria: Neosauropoda) from the 'middle' Cretaceous of central Patagonia, Argentina. *Journal of Vertebrate Paleontology* **31**, 93–110 (2011).

43. Carballido J, Salgado L, Pol D, Canudo J, Garrido A. A new basal rebbachisaurid (Sauropoda, Diplodocoidea) from the Early Cretaceous of the Neuquén Basin; evolution and biogeography of the group. *Historical Biology* **24**, 631–654 (2012).

44. Carballido JL, Pol D, Parra-Ruge ML, Padilla-Bernal S, Páramo-Fonseca ME, Etayo-Serna F. A new Early Cretaceous brachiosaurid (Dinosauria, Neosauropoda) from northwestern Gondwana (Villa de Leiva, Colombia). *Journal of Vertebrate Paleontology* **35**, e980505 (2015).

45. Tschopp E, Mateus O. Osteology of *Galeamopus pabsti* sp. nov. (Sauropoda: Diplodocidae), with implications for neurocentral closure timing, and the cervico-dorsal transition in diplodocids. *PeerJ* **5**, e3179 (2017).

46. Tschopp E, Mateus O. The skull and neck of a new flagellicaudatan sauropod from the Morrison Formation and its implication for the evolution and ontogeny of diplodocid dinosaurs. *Journal of Systematic Palaeontology* **11**, 853–888 (2013).

47. Carballido JL, Pol D. The dentition of *Amygdalodon patagonicus* (Dinosauria: Sauropoda) and the dental evolution of basal sauropods. *Comptes Rendus Palevol* **9**, 83-93 (2010).

48. Goloboff PA, Farris J, Nixon KC. TNT, a free program for phylogenetic analysis. *Cladistics* **24**, 774-786 (2008).

49. Goloboff PA, Farris JS, Källersjö M, Oxelman B, Ramírez M, Szumik CA. Improvements to resampling measures of group support. *Cladistics* **19**, 324-332 (2003).

50. Author. Mesquite: a modular system for evolutionary analysis (Version 2.75). http://mesquiteproject.org (2011).

51. Author. PAUP: phylogenetic analysis using parsimony version 4.10b10. . Sinauer Associates (2002).

52. Goloboff PA. Extended implied weighting. *Cladistics* **30**, 260–272 (2014).

53. Upchurch P, Martin J. The anatomy and taxonomy of Cetiosaurus (Saurischia: Sauropoda) from the Middle Jurassic of England. *Journal of Vertebrate Paleontology* **23**, 208–231 (2003).

54. Harris JD. The significance of Suuwassea emilieae (Dinosauria: Sauropoda) for flagellicaudatan intrarelationships and evolution. *Journal of Systematic Palaeontology* **4**, 185–198 (2006).

55. Berman DS, McIntosh JS. Skull and relationships of the Upper Jurassic sauropod Apatosaurus (Reptilia: Saurischia). *Bulletin of Carnegie Museum of Natural History* **8**, 1–35 (1978).

56. Charig AJ. A diplodocid sauropod from the Lower Cretaceous of England. In: *Aspects of Vertebrate History: Essays in Honor of Edwin Harris Colbert* (eds Jacobs L) 231–244 (Museum of Northern Arizona Press, 1980).

57. Gabunia LK, McHedlidze G, Chkhikvadze VM, Lucas SG. Jurassic sauropod dinosaur from the Republic of Georgia. *Journal of Vertebrate Paleontology* **18**, 233-236 (1998).

58. Upchurch P. Evolutionary history of sauropod Dinosaurs. *Philosophical Transactions of the Royal Society of London Series B-Biological Sciences* **349**, 365-390 (1995).

59. Day JJ, Upchurch P, Norman DB, Gale AS, P. PH. Sauropod trackways, evolution, and behavior. *Science* **296**, 1659 (2002).

60. D'Emic MD. The early evolution of titanosauriform sauropod dinosaurs. *Zoological Journal of the Linnean Society* **166**, 624–671 (2012).

61. Royo-Torres R, Upchurch P. The cranial anatomy of the sauropod Turiasaurus riodevensis and implications for its phylogenetic relationships. *Journal of Systematic Palaeontology* **10**, 553–583 (2012).

62. Wilson JA. Redescription of the mongolian sauropod *Nemegtosaurus mongoliensis* Nowinski (Dinosauria:Saurischia) and comments on Late Cretaceous sauropod diversity. *Journal of Systematic Palaeontology* **3**, 283-318 (2005).

63. Wilson JA, Upchurch P. Redescription and reassessment of the phylogenetic affinities of *Euhelopus zdanskyi* (Dinosauria: Sauropoda) from the Early Cretaceous of China. *Journal Systematic Palaeontology* **7**, 199–239 (2009).

64. Ouyang H. A new sauropod dinosaur from Dashanpu, Zigong County, Sichuan Province (*Abrosaurus dongpoensis* gen. et sp. nov.). *Newsletter of the Zigong Dinosaur Museum* **2**, 10–14 (1989).

65. Wilson JA, Sereno PC. Early evolution and higher-level phylogeny of sauropod dinosaurs. *Society of Vertebrate Paleontology Memoir* **5**, 1-68 (1998).

66. Upchurch P. The sauropodomorph supermatrix: towards a global phylogeny of the largest terrestrial animals. *Journal of Vertebrate Paleontology* **29(3: Suppl.)**, 194A (2009).

67. Monbaron M, Russell DA, Taquet P. *Atlasaurus imelakei* n. g., n. sp., a brachiosaurid-like sauropod from the Middle Jurassic of Morocco. *Comptes Rendus de l'Academie des Sciences, Paris* **329**, 519–526 (1999).

68. Royo-Torres R, Cobos A, Alcalá L. A giant European dinosaur and a new sauropod clade. *Science* **314**, 1925–1927 (2006).

69. Xing L*, et al.* A new sauropod dinosaur from the Late Jurassic of China and the diversity, distribution, and relationships of mamenchisaurids. *Journal of Vertebrate Paleontology* **35**, e889701 (2015).

70. Bonaparte JF. The early radiation and phylogenetic relationships of the Jurassic sauropod dinosaurs, based on vertebral anatomy. In: *The Beginning of the Age of Dinosaurs* (eds Padian K) 247–258 (Cambridge University Press, 1986).

71. McIntosh JS. Sauropoda. In: *The Dinosauria (First edition)* (eds Weishampel DB, Dodson P, Osmolska H) 345–401 (University of California Press, 1990).

72. Upchurch P. The phylogenetic relationships of sauropod dinosaurs. *Zoological Journal of the Linnean Society* **124**, 43–103 (1998).

73. Janensch W. Die gliedmaszen und gliedmaszengürtel der Sauropoden der Tendaguru-Schichten. *Palaeontographica (Supplement 7)* **3**, 177–235 (1961).

74. Läng É, Goussard F. Redescription of the wrist and manus of ?*Bothriospondylus madagascariensis*: new data on carpus morphology in Sauropoda. *Geodiversitas* **29**, 549–560 (2007).

75. Rauhut OWM, Lopez-Arbarello A. Archosaur evolution during the Jurassic: a southern perspective. *Revista de la Asociacion Geologica Argentina* **63**, 557–585 (2008).

76. Läng É, Mahammed F. New anatomical data and phylogenetic relationships of Chebsaurus algeriensis (Dinosauria, Sauropoda) from the Middle Jurassic of Algeria. *Historical Biology* **22**, 142–164 (2010).

77. Day JJ, Norman DB, Gale AS, Upchurch P, Powell HP. A Middle Jurassic dinosaur trackway site from Oxfordshire, UK. *Palaeontology* **47**, 319–348 (2004).

78. Wilson JA, Carrano MT. Titanosaurs and the origin of "wide-gauge" trackways: a biomechanical and systematic perspective on sauropod locomotion. *Paleobiology* **25**, 252–267 (1999).

79. Gilmore C. Reptilian fauna of the North Horn Formation of central Utah. . *United States Department of the Interior Geological Survey Professional Paper* **210-C**, 29–53 (1946).

80. Borsuk-Bialynicka M. A new camarasaurid sauropod *Opisthocoelicaudia skarzynskii* gen. n., sp. n. from the Upper Cretaceous of Mongolia. *Palaeontologica Polonica* **37**, 5–63 (1977).

81. Salgado L, Coria RA, Heredia S. Evolution of titanosaurid sauropods. I: phylogenetic analysis based on the postcranial evidence. *Ameghiniana* **34**, 3-32 (1997).

82. Henderson D. Burly gaits: centers of mass, stability and the trackways of sauropod dinosaurs. *Journal of Vertebrate Paleontology* **26**, 907–921 (2006).

83. Santos V, Moratalla J, Royo-Torres R. New sauropod trackways from the Middle Jurassic of Portugal. *Acta Palaeontologica Polonica* **54**, 409–422 (2009).

84. Dong Z. Sauropoda from the Kelameili Region of the Junggar Basin, Xinjiang Autonomous Region. *Vertebrata PalAsiatica* **28**, 43–58 (1990).

85. Clark JM, *et al.* The Mid-Late Jurassic terrestrial transition: new discoveries form the Shishugou Formation, China. In: *Ninth International Symposium on Mesozoic Terrestrial Ecosystems and Biota* (eds Barrett PM, Batten DJ, Evans S, Nudds J, Selden PA, Ross A) 26-28 (Department of Palaeontology, Natural History Museum of London, 2006).

86. Choiniere J, Clark J, Forster C, Xu X. A basal coelurosaur (Dinosauria: Theropoda) from the Late Jurassic (Oxfordian) of the Shishugou Formation in Wucaiwan, People's Republic of China. *Journal of Vertebrate Paleontology* **30**, 1773–1796 (2010).

87. Mo J. *Bellusaurus sui* Henan Science and Technology Press (2013).

88. Moore AJ, Mo J, Clark J, Xu X. New cranial material of Bellusaurus Sui (Dinosauria: Sauropoda) from the Middle-Late Jurassic Shishugou Formation of China supports neosauropod affinities. *Journal of Vertebrate Paleontology*, 183 (2015).

89. Mannion PD, Allain R, Moine O. The earliest known titanosauriform sauropod dinosaur and the evolution of Brachiosauridae. *PeerJ* **5**, e3217 (2017).

90. Upchurch P, Mannion PD. The first diplodocid from Asia and its implications for the evolutionary history of sauropod dinosaurs. *Palaeontology* **52**, 1195-1207 (2009).

91. Whitlock J, D'Emic M, Wilson J. Cretaceous diplodocids in Asia? Re-evaluating the phylogenetic affinities of a fragmentary specimen. *Palaeontology* **54**, 351–364 (2011).

92. Shimizu I, Chanthasit P, Suteethorn S, Sashida K, Agematsu S. New sauropod material from the Early Cretaceous of Thailand. *Journal of Vertebrate Paleontology*, 223 (2016).

93. Matzke NJ. Probabilistic historical biogeography: new models for founder-event speciation, imperfect detection, and fossils allow improved accuracy and model-testing. *Frontiers of Biogeography* **5**, 242–248 (2013).

94. Matzke NJ. Model selection in historical biogeography reveals that founder-event speciation is a crucial process in Island Clades. *Systematic Biology* **63**, 951–970 (2014).

95. Loewen MA, Irmis RB, Sertich JJW, Currie PJ, Sampson SD. Tyrant dinosaur evolution tracks the rise and fall of Late Cretaceous oceans. *PLOS ONE* **8**, 1-14 (2013).

96. Remes K. Revision of the Tendaguru sauropod dinosaur Tornieria africana (Fraas) and its relevance for sauropod paleobiogeography. *Journal of Vertebrate Paleontology* **26**, 651–669 (2006).

97. Lovelace DM, Hartman SA, Wahl WR. Morphology of a specimen of *Supersaurus* (Dinosauria, Sauropoda) from the Morrison Formation of Wyoming, and a re-evaluation of diplodocid phylogeny. *Arquivos do Museu Nacional* **65**, 527-544 (2007).

98. Gallina PA, Apesteguía S, Haluza A, Canale JI. A diplodocid sauropod survivor from the Early Cretaceous of South America. *PLoS ONE* **9**, e97128 (2014).

99. Bell MA, Lloyd GT. Strap: an R package for plotting phylogenies against stratigraphy and assessing their stratigraphic congruence. *Palaeontology* **58**, 379–389 (2015).

100. Author. R: A language and environment for statistical computing. R Foundation for Statistical Computing (2015).

101. Brusatte SL, Benton MJ, Ruta M, Lloyd GT. The first 50 Myr of dinosaur evolution: macroevolutionary pattern and morphological disparity. *Biology Letters* **4**, 733–736 (2008).

102. Bapst DW. A stochastic rate-calibrated method for time-scaling phylogenies of fossil taxa. *Methods in Ecology and Evolution* **4**, 724–733 (2013).

103. Poropat SF*, et al.* New Australian sauropods shed light on Cretaceous dinosaur palaeobiogeography. *Scientific Reports* **6**, 34467 (2016).

104. Seton M*, et al.* Global continental and ocean basin reconstructions since 200 Ma. *Earth-Science Reviews* **113**, 212–270 (2012).

105. Reeves C. The position of Madagascar within Gondwana and its movements during Gondwana dispersal. *Journal of African Earth Sciences* **94**, 45–57 (2014).

106. Ratheesh-Kumar RT, Ishwar-Kumar C, Windley Bf, Razakamanana T, Nair R, Sajeev K. India–Madagascar paleo-fit based on flexural isostasy of their rifted margins. *Gondwana Research* **28**, 581-600 (2016).

107. Baraboshkin EY, Alekseev AS, Kopaevich LF. Cretaceous palaeogeography of the North-Eastern Peri-Tethys. *Palaeogeography, Palaeoclimatology, Palaeoecology* **196**, 177–208 (2003).

108. Csiki-Sava Z, Buffetaut E, Ősi A, Pereda-Suberbiola X, Brusatte SL. Island life in the Cretaceous – faunal composition, biogeography, evolution, and extinction of land-living vertebrates on the Late Cretaceous European archipelago. *ZooKeys* **469**, 1–161 (2015).

109. Wierzbowski H, Rogov MA, Matyja Ba, Kiselev D, Ippolitov A. Middle–Upper Jurassic (Upper Callovian–Lower Kimmeridgian) stable isotope and elemental records of the Russian Platform: Indices of oceanographic and climatic changes. . *Global and Planetary Change* **107**, 196–212 (2013).

110. Colpaert C, Nikitenko B, Khafaeva S, Wall AF. The evolution of Late Callovian to Early Kimmeridgian foraminiferal associations from the central part of the Russian Sea (Makar'yev section, Volga River Basin, Russia). *Palaeogeography, Palaeoclimatology, Palaeoecology* **451**, 97–109 (2016).

111. Dera G*, et al.* Nd isotope constraints on ocean circulation, paleoclimate, and continental drainage during the Jurassic breakup of Pangea *Gondwana Research* **27**, 1599–1615 (2015).

112. Baraboshkin EJB. Tethyan correlation of the Lower Cretaceous ammonite scales. *Moscow University Geology Bulletin* **59**, 9–20 (2004).

113. Smith AG, Smith DG, Funnell BM. *Altas of Mesozoic and Cenozoic Coastlines*. Cambridge University Press (1994).

114. Burnham KP, Anderson DR. *Model selection and multimodel inference: a practical information ‐ theoretic approach 2nd ed.* . Springer (2002).

115. Upchurch P. Gondwanan break-up – legacies of a lost world? *Trends in Ecology and Evolution* **23**, 229-236 (2008).

116. Upchurch P, Hunn CA, Norman DB. An analysis of dinosaurian biogeography: evidence for the existence of vicariance and dispersal patterns caused by geological events. *Proc R Soc Lond Ser B-Biol Sci* **269**, 613-621 (2002).

117. Russell DA. The role of Central Asia in dinosaurian biogeography. *Canadian Journal of Earth Sciences* **30**, 2002-2012 (1993).

118. Le Loeuff J. Biogeography. In: *Encyclopedia of dinosaurs* (eds Currie PJ, Padian K) 51-56 (Academic Press, 1997).

119. Barrett PM, Hasegawa Y, Manabe M, Isaji S, Matsouka H. Sauropod dinosaurs from the Lower Cretaceous of eastern Asia: taxonomic and biogeographical implications. *Palaeontology* **45**, 1197–1217 (2002).
