# Supplementary material for: A new Middle Jurassic diplodocoid suggests an earlier dispersal and diversification of sauropod dinosaurs
Source: Nat Commun. 2018 Jul 24;9:2700. doi: 10.1038/s41467-018-05128-1 (PMC6057878; doi:10.1038/s41467-018-05128-1)
Supplement: Supplementary file 3 — Description of Additional Supplementary Files [file 41467_2018_5128_MOESM3_ESM.pdf]

# Description of Additional Supplementary Files

**File Name:** Supplementary Data 1
**Description:** Main Data matrix (tnt format).

**File Name:** Supplementary Data 2
**Description:** Subsidiary Data matrix (tnt format).

**File Name:** Supplementary Data 3
**Description:** Taxon age file.

**File Name:** Supplementary Data 4
**Description:** Time-calibrated phylogeny file.

**File Name:** Supplementary Data 5
**Description:** Taxon geographic range file.

**File Name:** Supplementary Data 6
**Description:** Dispersal multiplier matrices (relaxed).

**File Name:** Supplementary Data 7
**Description:** Dispersal multiplier matrices (harsh).

**File Name:** Supplementary Data 8
**Description:** Time periods file.

**File Name:** Supplementary Data 9
**Description:** R script for BioGeoBears.

**File Name:** Supplementary Data 10
**Description:** BioGeoBEARS ancestral area estimation plots (relaxed).

**File Name:** Supplementary Data 11
**Description:** BioGeoBEARS ancestral area estimation plots (harsh).

**File Name:** Supplementary Data 12
**Description:** Time-calibrated tree used in reduced BioGeoBEARS analysis.

**File Name:** Supplementary Data 13
**Description:** reduced data set BioGeoBEARS ancestral area estimation plots (relaxed).

**File Name:** Supplementary Data 14
**Description:** reduced data set BioGeoBEARS ancestral area estimation plots (harsh).

**File Name:** Supplementary Data 15
**Description:** Summary of the synapomorphies supporting the placement of Lingwulong within Diplodocoidea, Flagellicaudata, Dicraeosauridae, and the node uniting Lingwulong and 'higher' dicraeosaurids (i.e., Amargasaurus, Brachytrachelopan, and Dicraeosaurus). Only characters that can be scored for Lingwulong are listed here. Character mapping was carried out based on the main and subsidiary datasets for the trees in Supplementary Figs. 13 and 15 respectively, using Mesquite vs. 2.75 50. 'M' and 'S' identify character numbers and synapomorphies obtained from the main and subsidiary datasets, respectively.

**File Name:** Supplementary Data 16
**Description:** Full dataset. Summary of results and statistical comparisons between the six biogeographic models applied in the BioGeoBEARS analyses. The 'Ratio' in the AIC analyses is the ratio of the AIC weight for the +J version of the same model (e.g., DEC+J/DEC). An asterisk (*) marks those models that are regarded as best fitting the data in each analysis (see Supplementary Note 6 for details).

**File Name:** Supplementary Data 17
**Description:** Reduced dataset (i.e., with Tornieria, Supersaurus, Dinheirosaurus, and Leinkupal omitted). Summary of results and statistical comparisons between the six biogeographic models applied in the BioGeoBEARS analyses. The 'Ratio' in the AIC analyses is the ratio of the AIC weight for the +J version of the same model (e.g., DEC+J/DEC). An asterisk (*) marks those models that are regarded as best fitting the data in each analysis (see Supplementary Note 6 for details).
