# Supplementary figures and images for: A new Middle Jurassic diplodocoid suggests an earlier dispersal and diversification of sauropod dinosaurs
Source: Nat Commun. 2018 Jul 24;9:2700. doi: 10.1038/s41467-018-05128-1 (PMC6057878; doi:10.1038/s41467-018-05128-1)

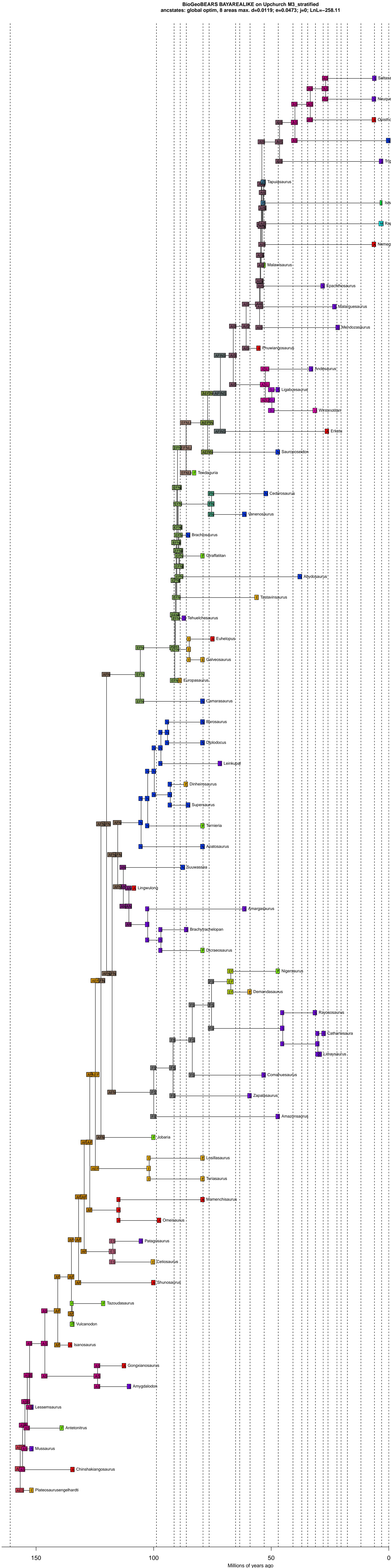

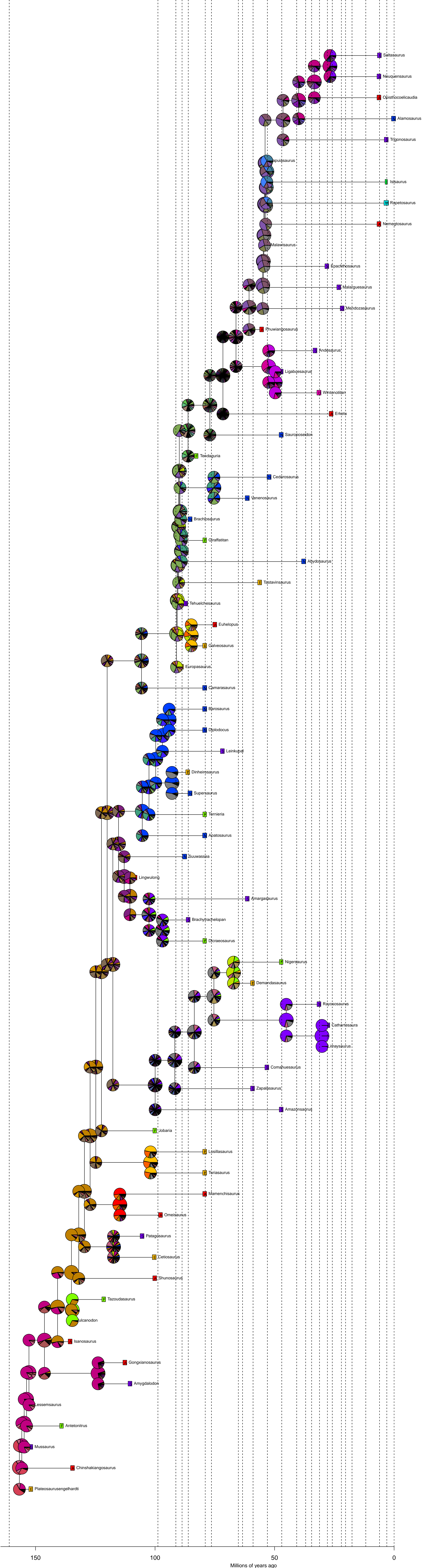

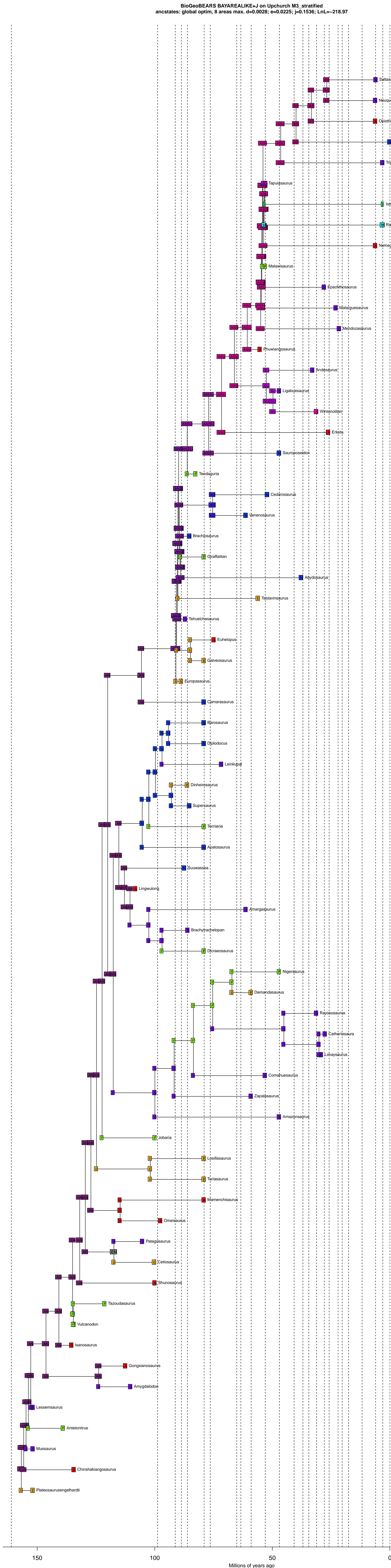

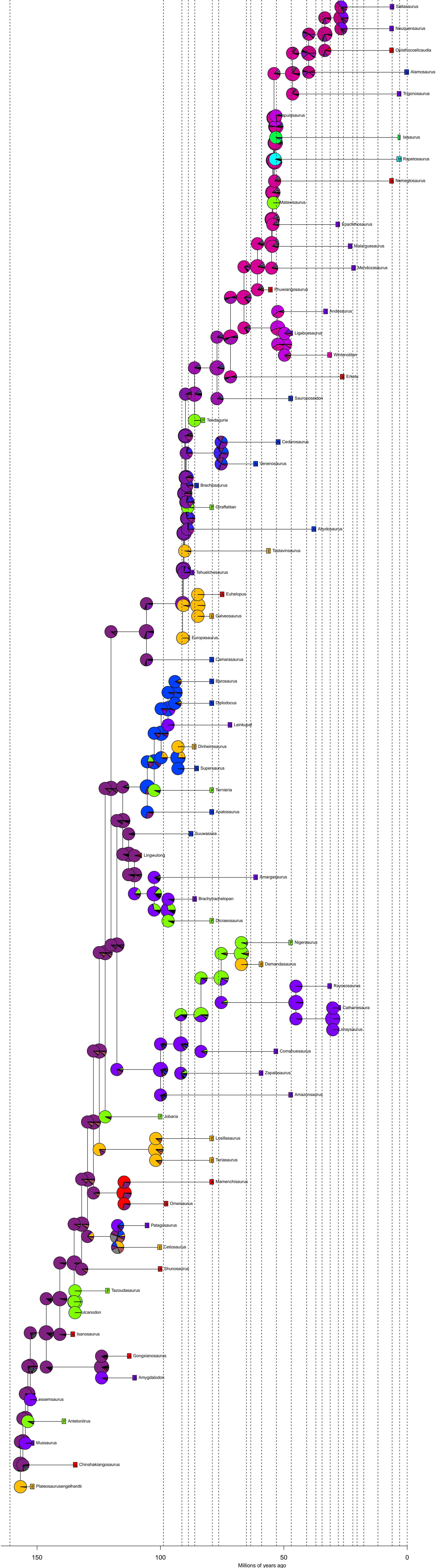

Supplement: Supplementary file 13 — Supplementary Data 10 [file 41467_2018_5128_MOESM13_ESM.pdf]

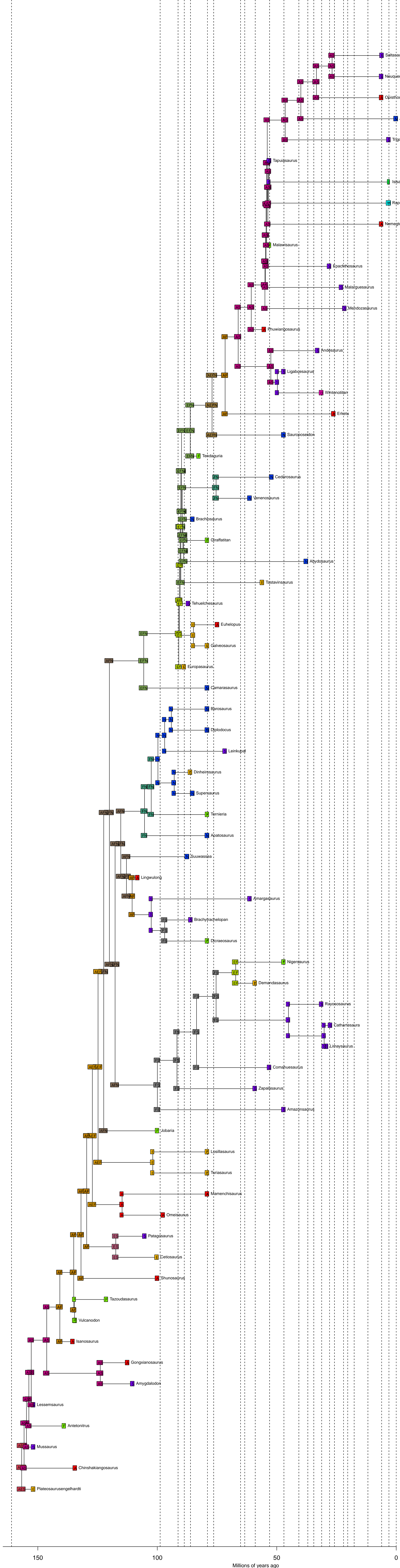

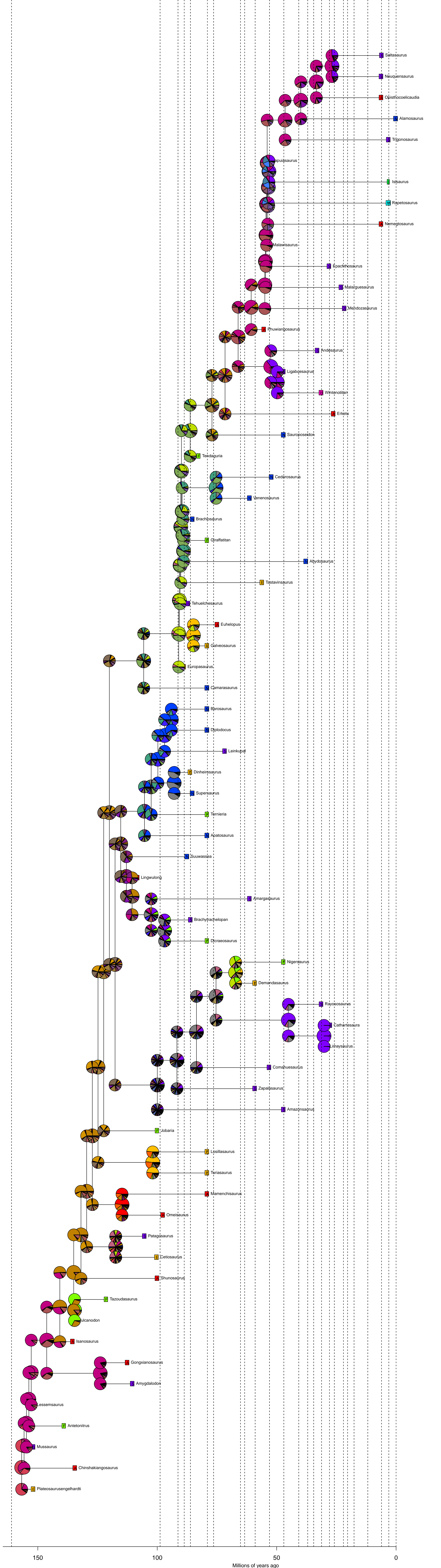

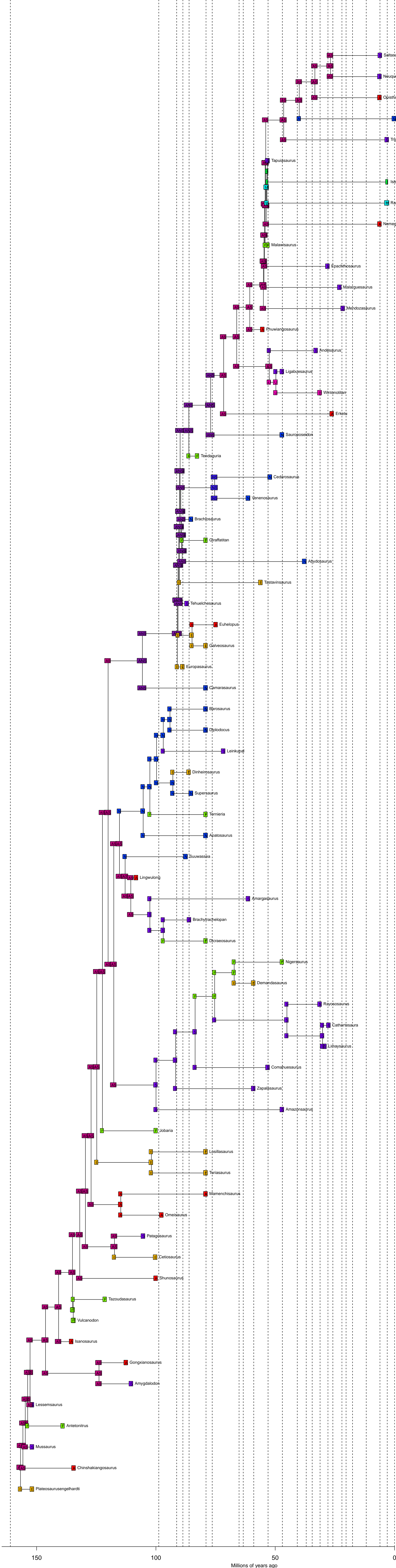

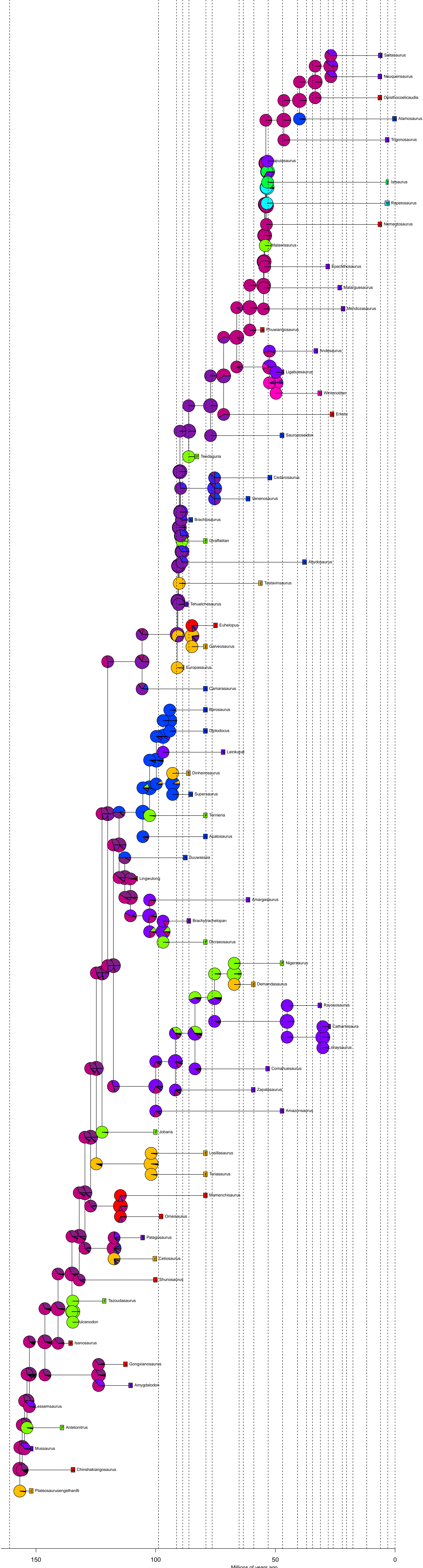

Supplement: Supplementary file 14 — Supplementary Data 11 [file 41467_2018_5128_MOESM14_ESM.pdf]

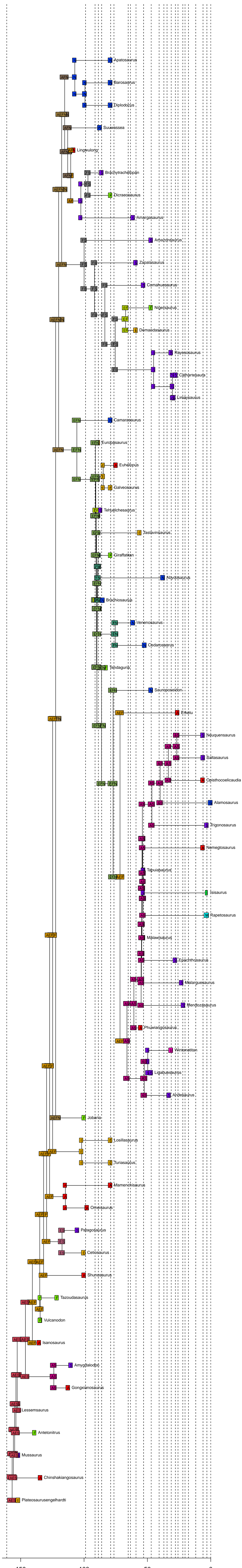

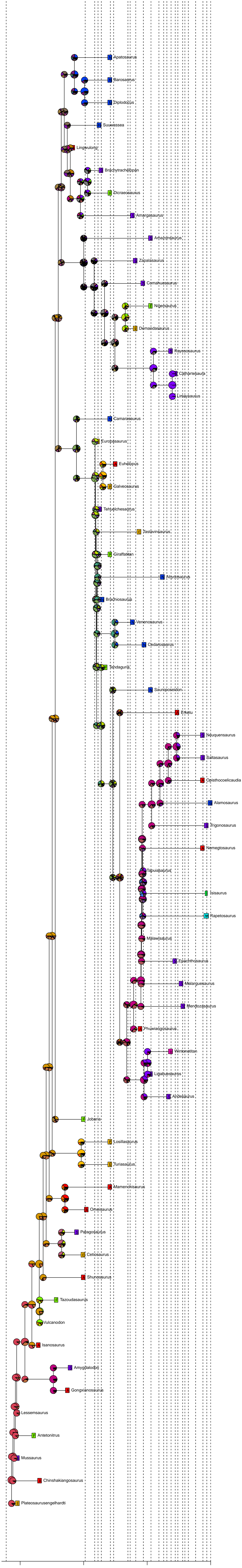

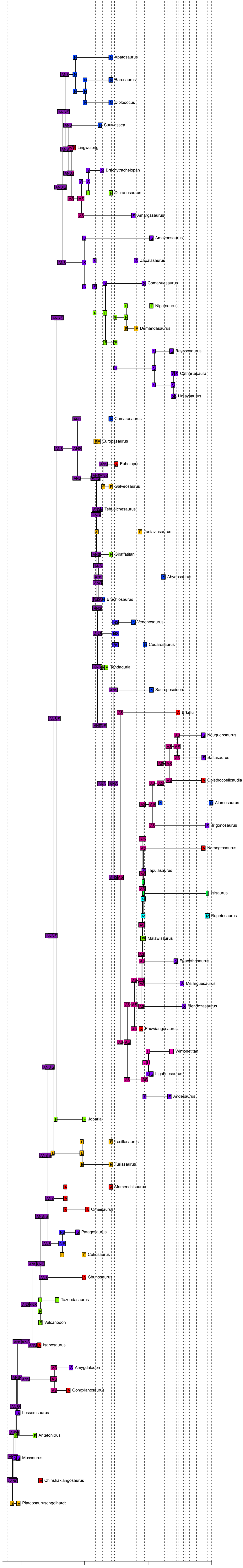

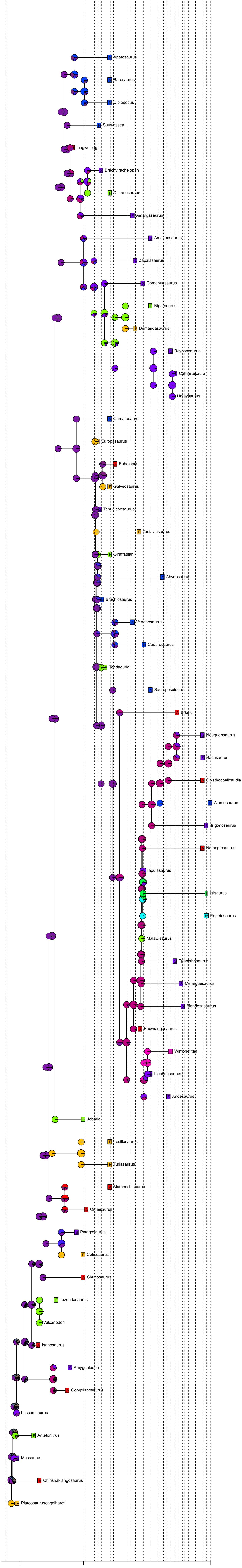

Supplement: Supplementary file 16 — Supplementary Data 13 [file 41467_2018_5128_MOESM16_ESM.pdf]

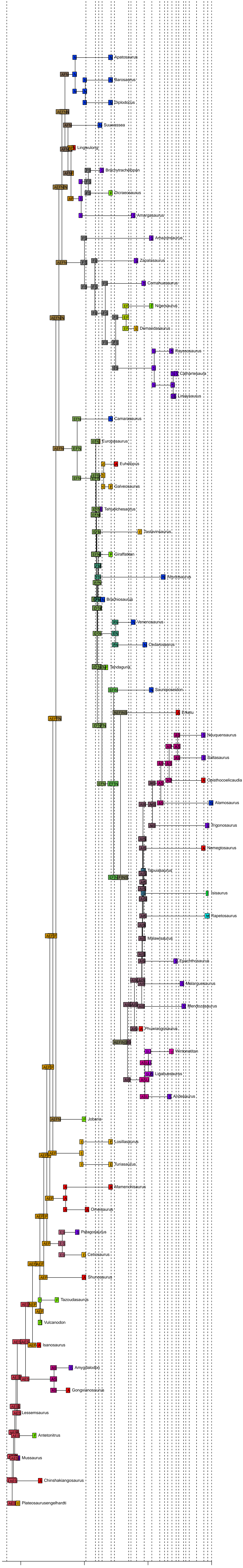



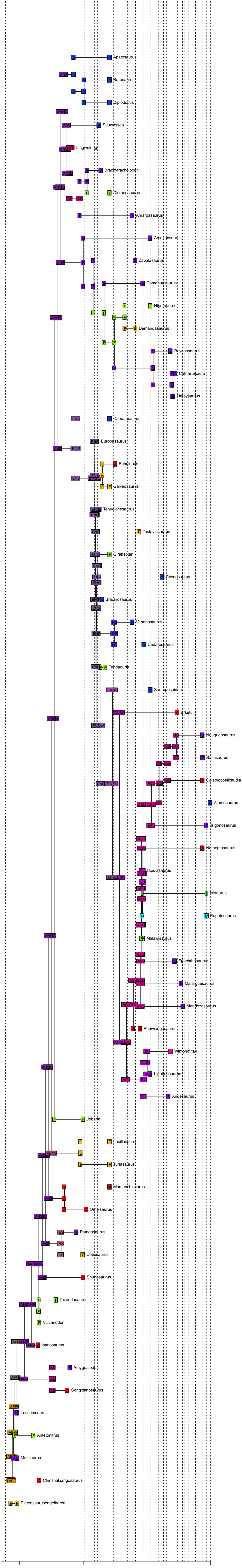

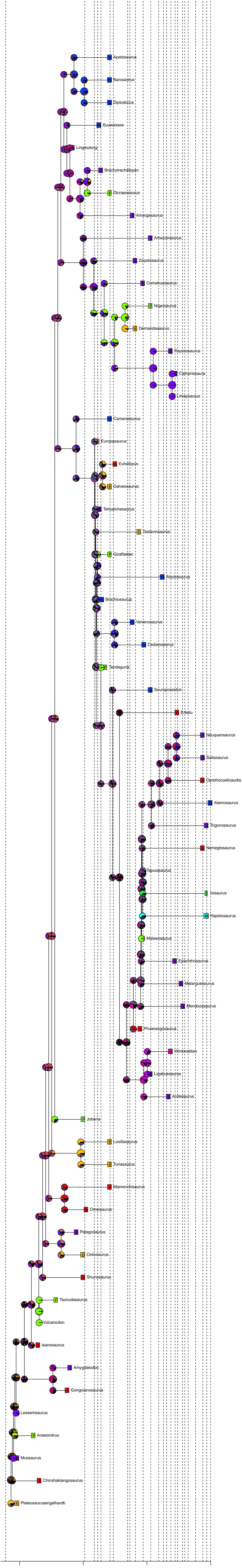

Supplement: Supplementary file 17 — Supplementary Data 14 [file 41467_2018_5128_MOESM17_ESM.pdf]
